# Effect of ChCl:Glycolic Acid Molar Ratio and Plant Extract Incorporation on the Structure–Property Relationships of Chitosan Active Packaging Films

**DOI:** 10.3390/polym18202459

**Published:** 2026-10-09

**Authors:** Paul Stanescu, Adina Gavrila, Gabriela Isopencu, Ludmila Motelica, Adrian Ionut Nicoara, Sergiu Sima, Mihaela Bumbaru Dandes, Oana Ciocirlan, Ovidiu-Cristian Oprea

**Affiliations:** 1 Faculty of Chemical Engineering and Biotechnologies, National University of Science and Technology POLITEHNICA Bucharest, 1–7 Gh. Polizu Street, 011061 Bucharest, Romania; paul.stanescu@upb.ro (P.S.); adina.gavrila@upb.ro (A.G.); gabriela.isopencu@upb.ro (G.I.); adrian.nicoara@upb.ro (A.I.N.); sergiu.sima@upb.ro (S.S.); mihaela.bumbaru@gmail.com (M.B.D.); ovidiu.oprea@upb.ro (O.-C.O.); 2 Research Center for Advanced Materials, Products and Processes, National University of Science and Technology POLITEHNICA Bucharest, 313 Splaiul Independentei, 060042 Bucharest, Romania; ludmila.motelica@upb.ro; 3 National Research Center for Micro and Nanomaterials, National University of Science and Technology POLITEHNICA Bucharest, 313 Spl. Independenței, 060042 Bucharest, Romania; 4 Academy of Romanian Scientists, 3 Ilfov Street, 050044 Bucharest, Romania

**Keywords:** chitosan, NaDES, choline chloride, glycolic acid, plant extracts, antioxidant activity, active packaging

## Abstract

The influence of choline chloride:glycolic acid (ChCl:GA) stoichiometry and plant extract incorporation on the properties of chitosan-based active packaging films was investigated. A deep eutectic solvent with a ChCl:GA molar ratio of 1:2 (DES_GA2) was used both as a chitosan plasticizer and an extraction medium for neem and hawthorn compounds. Films containing 44–70 wt% DES_GA2 were characterized to establish composition–structure–property relationships. The 50 wt% formulation showed a favorable balance between mechanical flexibility and water vapor barrier performance and was selected for plant extract incorporation. Neem extract showed higher radical-scavenging activity than hawthorn despite its lower total phenolic content. Extract incorporation enhanced UV shielding and reduced water vapor permeability, with the hawthorn-containing film showing an approximately 41% reduction in WVP. Extract-containing films retained antioxidant functionality and showed antibacterial activity against *Escherichia coli*. Comparison with the previously investigated ChCl 1:3 system revealed stoichiometry-dependent behavior. At 50 wt% NaDES, increasing the ChCl:GA molar ratio to 1:2 increased elongation at break from 90.5% to 120.1% and reduced Young’s modulus from 131.0 to 48.0 MPa. Comparison of neem-loaded films based on different NaDES systems further demonstrated the influence of the NaDES composition. These results identify the NaDES composition and botanical source as relevant formulation variables for tailoring CS–NaDES active packaging films.

## 1. Introduction

Biodegradable polymers have increasingly been investigated as matrices for active food packaging, with the dual objective of extending food shelf life and reducing reliance on conventional petroleum-based plastics [1,2,3]. Among these polymers, chitosan (CS) is particularly attractive because of its film-forming ability, intrinsic antimicrobial activity, derivation from abundant chitin-containing biomass, and capacity to incorporate a variety of functional compounds [4,5,6,7,8,9]. Despite these advantages, neat chitosan films are generally rigid and brittle and exhibit limited resistance to water vapor, which restricts their use in food packaging [10,11]. Plasticizers are commonly incorporated into chitosan matrices to improve film flexibility, although their addition may also affect mechanical strength and water vapor barrier properties [12,13,14,15,16]. Plasticized chitosan materials can be prepared through wet processing, typically involving dissolution followed by solution casting, or through dry thermomechanical processing with little or no added solvent. Wet processing is uniquely suitable for active film formulations because plasticization and the incorporation of functional compounds can be achieved within the same film-forming solution [17,18].

In this context, natural deep eutectic solvents (NaDESs) are particularly interesting because they can fulfill two complementary roles in the development of active chitosan films: as plasticizers capable of increasing polymer chain mobility and film flexibility [19,20,21] and as extraction media for phytochemicals with antioxidant and antimicrobial activity [22,23,24,25,26,27]. More recently, these two functions have been combined by incorporating NaDES-based plant extracts directly into chitosan films, thereby coupling matrix plasticization with the introduction of bioactive functionality [28,29,30,31,32].

Acidic NaDESs based on choline chloride (ChCl) combined with organic hydrogen bond donors (lactic acid, glycolic acid, and others) are particularly relevant for chitosan plasticization. In ChCl-based acidic NaDESs, interactions with chitosan involve both hydrogen bonding and ionic interactions with protonated amino groups, disrupting native interchain interactions and increasing polymer-chain mobility, thereby promoting plasticization [33,34,35,36]. The relevance of such ionic and hydrogen bonding interactions has also been demonstrated in solvation studies of chitosan-derived species in ionic media, supported by both experimental measurements and DFT calculations [37].

An important but underexplored dimension of NaDES design is the role of the HBA:HBD molar ratio in governing both the physicochemical properties of the solvent and its subsequent effects on the polymer matrix. The physicochemical properties of ChCl-based eutectic systems, particularly density and viscosity, are strongly influenced by composition and temperature [38]. More specifically, variations in HBA:HBD stoichiometry can modify the hydrogen-bond network, acidity, and viscosity of NaDESs [39,40,41], thereby affecting polymer–plasticizer interactions and, consequently, the mechanical and barrier properties of the resulting films. This aspect has received comparatively little systematic attention compared to the choice of NaDES components [35].

Our recent work demonstrated that a ChCl:GA NaDES at a 1:3 molar ratio can function as both a plasticizer and an extraction medium for hawthorn polyphenols in chitosan films, with the 50 wt% formulation providing the most favorable balance between mechanical flexibility and barrier properties [42]. Whether this balance of properties is specific to the 1:3 stoichiometry or varies with the ChCl:GA molar ratio remains an open question.

The present study addresses this question using a wet-processing approach based on solution casting by systematically examining the ChCl:GA NaDES with a lower glycolic acid content (1:2 molar ratio, DES_GA2) than the previously investigated 1:3 system (DES_GA3) [42]. By preparing and characterizing CS films in the same concentration range (44–70 wt% DES_GA2) used previously for the 1:3 system, the study enables assessment of the influence of NaDES stoichiometry on composition–structure–property relationships. In addition, hawthorn (*Crataegus monogyna* L.) was retained as a reference botanical source in order to enable direct comparison with the prior system, while neem (*Azadirachta indica*), a chemically and functionally distinct polyphenol source [24,43], was introduced in order to examine the effect of botanical identity on film functionality. A ChCl:lactic acid (1:1) neem-loaded film was included as an additional comparative system to assess how the NaDES medium influences the response to the same botanical extract.

The novelty of this work lies in three main aspects: (i) the effect of ChCl:GA stoichiometry on chitosan film properties is examined by comparing the 1:2 system with the previously studied 1:3 system; (ii) the extraction and functional effects of two chemically distinct plant sources, hawthorn and neem, are compared using the same NaDES; and (iii) the influence of NaDES composition on extract-containing films is explored by comparing neem-containing films based on a ChCl:GA and a ChCl:LA NaDES. The density, viscosity, and FTIR characteristics of DES_GA2 are also reported in order to provide a physicochemical context for the observed film behavior.

## 2. Materials and Methods

### 2.1. Materials

Glycolic acid (GA, ≥99 wt%; Carlo Erba, Cornaredo, Italy) and choline chloride (ChCl, ≥98 wt%; Thermo Scientific Chemicals, Waltham, MA, USA) were used as received. Chitosan (ACRO34905, MW = 100–300 kDa, degree of deacetylation ≥75%; Thermo Scientific Chemicals) was likewise used without further purification. 1,1-Diphenyl-2-picrylhydrazyl (DPPH), gallic acid, ethanol (96%), and sodium carbonate were sourced from Sigma-Aldrich, St. Louis, MO, USA; Folin–Ciocalteu reagent (analytical grade) was obtained from Merck (Darmstadt, Germany). Doubly distilled water was used throughout for the preparation of film-forming and extraction solutions.

Neem (*Azadirachta indica*) leaves were supplied by SC Santo Raphael SA (Bucharest, Romania). Hawthorn (*Crataegus monogyna* L.) flowers and leaves were obtained from Fares^®^ (Laboratoarele Fares Biovital SRL, Orăștie, Romania) and correspond to the same commercial batch used in our previous study [42], ensuring botanical consistency. All dried plant materials were ground and sieved to a particle size below 315 μm prior to use.

### 2.2. Preparation of the NaDES

ChCl and GA were combined at a 1:2 molar ratio in a 50 mL vessel and stirred at 60 °C for 1 h until a clear, homogeneous liquid was obtained. The resulting NaDES was designated DES_GA2. By comparison, DES_LA1 (ChCl:lactic acid, 1:1), used in the comparative film formulation, was also prepared following a similar procedure.

### 2.3. Physicochemical Characterization of DES_GA2

Density and dynamic viscosity of DES_GA2 were measured over the temperature range 20–60 °C using an Anton Paar DMA 4500 vibrating-tube densimeter, Anton Paar GmbH, Graz, Austria (±0.00005 g cm^−3^) and an Anton Paar AMVn automated microviscometer, Anton Paar GmbH, Graz, Austria, equipped with a calibrated 4.0 mm capillary (reproducibility <0.5%; repeatability ±0.1%). Both instruments operated under Peltier temperature control (±0.01 K). The FTIR spectrum of neat DES_GA2 was recorded using the conditions described in Section 2.10.1.

### 2.4. Preparation of Film-Forming Solutions

Film-forming solutions were prepared using the general CS–NaDES casting scheme previously reported [42], with the compositions adjusted to the DES_GA2 formulations investigated here. DES_GA2 contents of 44, 50, 60, and 70 wt% of the film solids were obtained by adjusting the relative amounts of CS and DES_GA2 while maintaining an approximately constant total solids concentration, as detailed in Table S1. At higher NaDES contents, the CS concentration was reduced because complete dissolution was not achievable under the preparation conditions. Chitosan was dissolved in the corresponding aqueous DES_GA2 solutions under continuous magnetic stirring at room temperature overnight. All extract-free solutions were clear and were filtered prior to casting to remove residual insoluble material.

For extract-containing films, 30% (*w*/*w*) aqueous NaDES solutions incorporating the relevant plant extract were used in place of the extract-free NaDES (Table S1). Specifically, 1.67 g of the 30% (*w*/*w*) aqueous NaDES extract solution was added to each formulation, corresponding to approximately 0.50 g of the NaDES. This amount was selected to maintain the NaDES content at 50 wt% of the film solids. Thus, the amount of extract solution incorporated was determined by the targeted NaDES content rather than by a predetermined dry-extract or phenolic loading. Hawthorn- and neem-containing films were formulated with DES_GA2, while an additional neem-containing film was prepared with DES_LA1 as a comparative system. The NaDES content was held at 50 wt% in all extract-containing formulations, with the CS concentration maintained at 1% (*w*/*v*). The extract-containing solutions contained visible dark particulates (Figure S1) and required vacuum filtration prior to casting owing to their higher viscosity (Figure S2).

The resulting films were designated 44DES_GA2, 50DES_GA2, 60DES_GA2, 70DES_GA2, 50DES_GA2_EN, 50DES_GA2_EH, and 50DES_LA1_EN, where EN and EH denote neem and hawthorn extracts, respectively.

### 2.5. Film Casting and Conditioning

Filtered film-forming solutions were poured into Petri dishes of identical diameter and dried at 50 °C overnight. Equal volumes of solution (approximately 2% *w*/*v* total solids) were cast in each dish to obtain comparable film thicknesses. After drying, the films were conditioned at room temperature and a relative humidity of 50 ± 2% until the time of analysis, then carefully peeled from the dishes before measurement.

### 2.6. Preparation of Plant Extracts

Aqueous solutions of 30% (*w*/*w*) NaDES were used as extraction media at a solvent-to-plant ratio of 20:1 (*v*/*w*). All extractions were performed at 50 °C for 10 min. Hawthorn compounds were extracted by microwave-assisted extraction (MAE) using a Biotage Initiator reactor (Biotage AB, Uppsala, Sweden) at 900 rpm and 12–27 W microwave power, following the procedure used in our previous study [42].

Neem (*Azadirachta indica*) extraction was performed using an ultrasonic probe (Vibra-Cell VCX750, Sonics & Materials, Newtown, CT, USA) coupled to a jacketed glass reactor set to 20% amplitude, with temperature and time controlled at 50 °C and 10 min, respectively, following the procedure described in our previous study [44]. Following each extraction, the resulting suspension was centrifuged at 4000 rpm for 10 min (MC5000, LBX Instruments, Barcelona, Spain); the supernatant was collected and stored refrigerated until analysis. Extracts are designated Ex_EH_GA2 (hawthorn/DES_GA2), Ex_EN_GA2 (neem/DES_GA2), and Ex_EN_LA1 (neem/DES_LA1).

### 2.7. Total Phenolic Content (TPC)

TPC was determined colorimetrically by the Folin–Ciocalteu method, as detailed in [45]. Each extract was diluted 25-fold with distilled water; 0.5 mL of the diluted sample was combined with 5 mL of 10% Folin–Ciocalteu reagent, stirred for 5 min, and treated with 1.5 mL of 20% sodium carbonate solution and 3 mL of distilled water. After 60 min of incubation in the dark at room temperature, absorbance was read at 760 nm (Shimadzu UVmini-1240, Kyoto, Japan). Results are expressed as mg gallic acid equivalents per gram of dry plant material (mg GAE g^−1^ DM) from a gallic acid calibration curve (1–5 mg mL^−1^). For comparative purposes, TPC was also determined for extracts prepared with 50% (*v*/*v*) aqueous ethanol.

### 2.8. DPPH Radical Scavenging Activity

Antioxidant activity was assessed by the DPPH radical scavenging assay following previously reported procedures [9,46], with minor modifications.

A stock DPPH solution (0.0071 mM; 0.280 mg DPPH dissolved in 100 mL ethanol) was prepared fresh for each assay. For film samples, 20 mg of film was cut into small pieces (ca. 2 × 2 mm) and was dispersed in 4 mL of distilled water and vortexed; 1 mL of the resulting suspension was mixed with 4 mL of DPPH solution and incubated in the dark at room temperature for 30 min. For extract samples, 1 mL of undiluted extract was combined directly with 4 mL of DPPH solution and incubated identically; after incubation, the mixture was centrifuged and the supernatant absorbance recorded. All absorbance measurements were made at 517 nm. DPPH scavenging activity (%) was calculated as

(1) $$\%DPPH=\frac{A^{DPPH}-A^{sample}}{A^{DPPH}}\times 100$$

where *A*^DPPH^ and *A*^sample^ are the absorbance of the DPPH solution and the sample-containing mixture, respectively. Measurements were performed in triplicate.

### 2.9. Antibacterial Activity

Antibacterial performance against *Escherichia coli* (DH5K; Gram-negative) and *Bacillus subtilis spizizenii* Nakamura (ATCC 6633; Gram-positive) was assessed by the agar disc-diffusion method. The bacterial inoculum was standardized to 0.6 McFarland; the corresponding optical densities were 0.2785 (*E. coli*) and 0.3015 (*B. subtilis*) for extract and extract-free film assays, and 0.1827 (*E. coli*) and 0.1735 (*B. subtilis*) for extract-loaded film assays. Agar plates were inoculated with 100 μL of suspension, allowed to dry for approximately 1 h, and then loaded with test samples. Extract-free films were assessed as approximately 2 × 2 cm squares; extract-loaded films and extract-soaked discs (6 μL per disc) were tested as 6 mm diameter discs in accordance with [47]. All film specimens were UV-sterilized at 254 nm for 30 min before placement. Plates were incubated at 37 °C for 24 h, and inhibition zones were measured.

### 2.10. Film Characterization

#### 2.10.1. FTIR Spectroscopy

Attenuated total reflectance (ATR)–FTIR spectra of films, a neat NaDES, and individual components were collected on a Nicolet iS50 spectrometer (Thermo Fisher Scientific, Waltham, MA, USA) over 4000–400 cm^−1^ at 2 cm^−1^ resolutions, averaging 32 scans per measurement. Spatially resolved FTIR maps were acquired with a Nicolet iS10MX FTIR microscope, Thermo Fisher Scientific Inc., Waltham, MA, USA (4000–650 cm^−1^).

#### 2.10.2. Thermogravimetric and Calorimetric Analysis

Simultaneous TG–DSC measurements were performed on a STA449C F3 Jupiter instrument (NETZSCH, Selb, Germany) from 20 to 800 °C under a dynamic airflow of 50 mL min^−1^.

#### 2.10.3. UV–Vis Absorption and Photoluminescence

UV–Vis spectra were recorded on a JASCO V-560 spectrophotometer (JASCO, Easton, PA, USA) equipped with a 60 mm integrating sphere (ISV-469) and a dedicated film holder, scanning from 200 to 900 nm at 200 nm min^−1^. Photoluminescence (PL) spectra were measured on a PerkinElmer LS55 spectrometer (PerkinElmer, Waltham, MA, USA) using a xenon lamp excitation source. Emission was collected from 350 to 800 nm at 200 nm min^−1^ (excitation and emission slit widths 10 nm; 350 nm cut-off filter; excitation wavelength 320 nm).

#### 2.10.4. Scanning Electron Microscopy

Surface morphology was examined by SEM using a Quanta Inspect F50 field-emission microscope (Thermo Fisher, Eindhoven, The Netherlands; spatial resolution 1.2 nm) with an Everhart–Thornley secondary electron detector at 30 kV accelerating voltage and a spot size of 3.5 at magnifications of 1000× and 10,000×.

#### 2.10.5. Tensile Testing

Mechanical properties were measured on an INSTRON universal testing machine, Darmstadt, Germany. Film strips (50 × 17 mm) were mounted between rubber-coated grips at a 20 mm gauge length and extended at 5 mm min^−1^ with a 0.05 N pre-tension. Young’s modulus (YM, MPa) was derived from the slope of the stress–strain curve over 0.05–2% strain; tensile strength (TS, MPa) and elongation at break (EB, %) were read directly from the curves. Film thickness was measured at five positions with a 0.001 mm-resolution micrometer. Triplicate measurements were performed.

#### 2.10.6. Moisture Content, Water Solubility, and Water Vapor Permeability

Moisture content (MC) and water solubility (WS) were determined gravimetrically on 20 × 20 mm specimens:

(2) $$MC\left(\%\right)=\frac{m_{0}-m_{1}}{m_{0}}\times 100$$

(3) $$WS\left(\%\right)=\frac{m_{1}-m_{2}}{m_{1}}\times 100$$

where *m*_0_ is the initial mass, *m*_1_ is the mass after drying at 105 °C to constant weight, and *m*_2_ is the dry mass recovered after 14 h immersion in 50 mL distilled water.

Water vapor permeability (WVP) was measured gravimetrically by sealing film specimens over glass vials containing anhydrous CaCl_2_ and placing the vials in an environment maintained at 75% relative humidity. The mass gain was recorded over 2–3 days, and WVP was calculated as

(4) $$WVP=\frac{∆m\cdot d}{∆t\cdot A\cdot P_{sat}\cdot ∆RH}$$

where Δ*m*/Δ*t* is the mass gain rate (g s^−1^), *d* is mean film thickness (m), *A* is exposed area (m^2^), *P_sat_* is the saturated water vapor pressure at the experimental temperature (Pa), and *DRH* is the relative humidity difference across the film, expressed as a decimal fraction. Under the experimental conditions, Δ*RH* = 0.75, assuming approximately 0% RH inside the vial due to the presence of anhydrous CaCl_2_ and 75% RH outside the vial. Measurements were performed in triplicate.

### 2.11. Statistical Analysis

The results of the mechanical, water vapor permeability (WVP), antioxidant, and antibacterial assays were expressed as mean ± standard deviation (SD). Differences among film formulations were evaluated by one-way analysis of variance (ANOVA) followed by Tukey’s honest significant difference (HSD) post hoc test. Differences were considered statistically significant at *p* < 0.05, corresponding to a 95% confidence level.

## 3. Results and Discussion

The discussion focuses on four complementary aspects: (i) the effect of DES_GA2 content on film structure and properties; (ii) the impact of plant extract incorporation on the selected 50DES_GA2 formulation; (iii) the influence of ChCl:GA stoichiometry, assessed through comparison with the previously characterized DES_GA3 system for both extract-free and hawthorn-containing films; and (iv) the influence of NaDES composition, assessed using neem as a common botanical source in DES_GA2- and DES_LA1-based films. The physicochemical characterization of DES_GA2 is provided in the Supplementary Information.

### 3.1. FTIR Spectral Analysis

#### 3.1.1. Effect of DES_GA2 Content 

**Figure 1:**
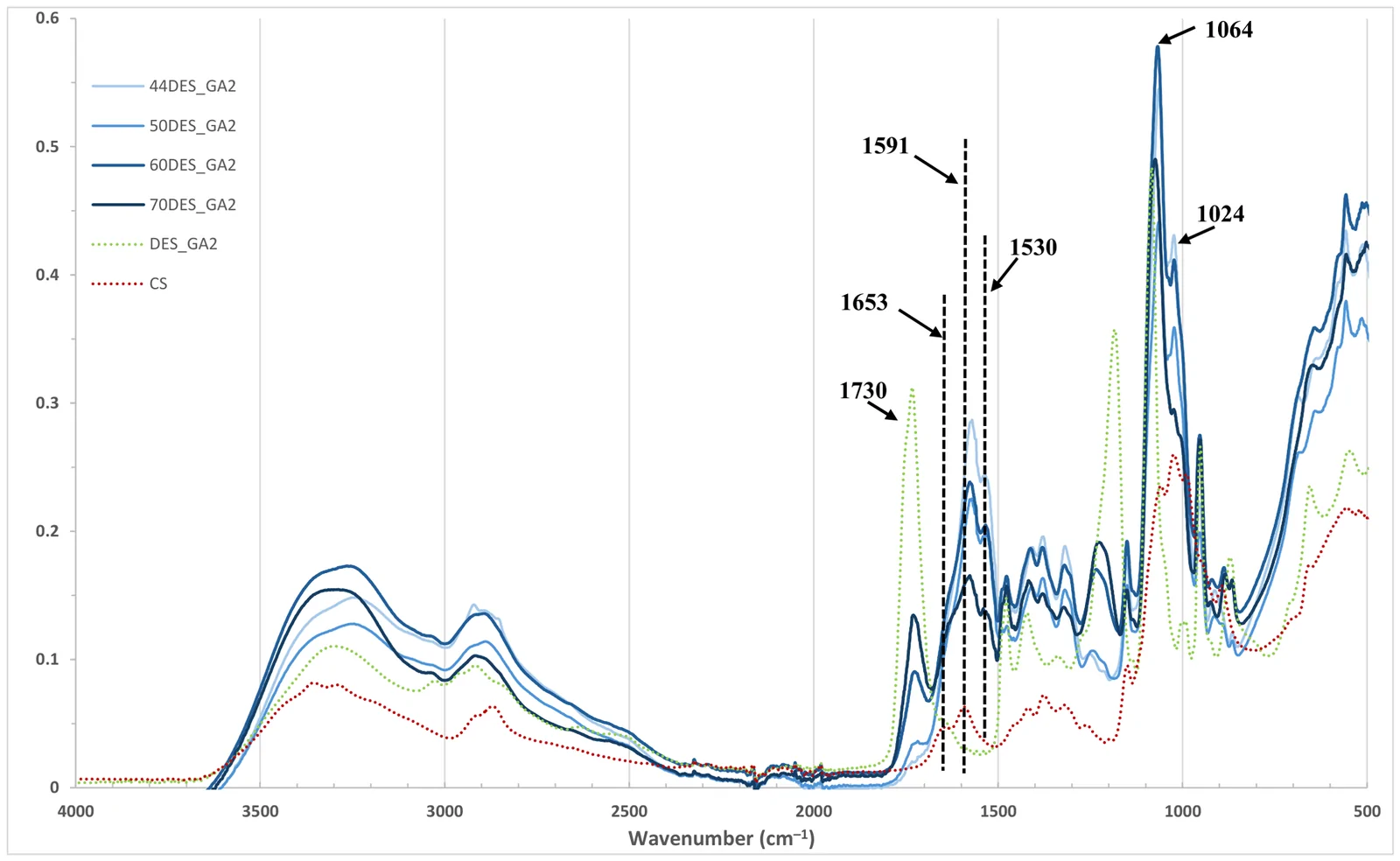
FTIR spectra of CS–NaDES films containing different DES_GA2 contents, together with neat CS and DES_GA2 as reference materials.

FTIR spectroscopy was used to probe the molecular interactions governing film structure as a function of NaDES content, as shown in Figure 1.

All CS–DES_GA2 films displayed a broad absorption band in the 3600–3000 cm^−1^ region attributable to overlapping O–H and N–H stretching contributions. The positional and intensity changes of this band relative to neat chitosan and pure DES_GA2 indicate substantial modification of the hydrogen bonding network upon film formation [48]. The N–H bending band of chitosan amino groups, observed at approximately 1591 cm^−1^ in the neat polymer, shifted and changed in profile in the films, with the apparent band maximum occurring at 1571–1580 cm^−1^. The absolute intensity of this region increased with decreasing DES_GA2 content (70DES_GA2 < 60DES_GA2 < 50DES_GA2 < 44DES_GA2); however, this trend also reflects the increasing chitosan content of the corresponding film-forming formulations and therefore cannot be directly interpreted as a quantitative measure of amino-group protonation.

To further evaluate these spectral changes, semi-quantitative peak fitting was performed in the 1500–1650 cm^−1^ region (Table S2 and Figure S3). Three overlapping components were resolved at approximately 1528–1529, 1574–1579, and 1621–1626 cm^−1^. The central component was predominant in all formulations, accounting for approximately 71–73% of the total fitted area, while its position progressively shifted from 1573 cm^−1^ for 44DES_GA2 to 1579 cm^−1^ for 70DES_GA2. These changes occurred concurrently with a progressive decrease in the pH of the film-forming solutions from 5.7 for 44DES_GA2 to 5.1, 3.9, and 3.4 for 50DES_GA2, 60DES_GA2, and 70DES_GA2, respectively (Table S1). The increasingly acidic environment favors protonation of the chitosan amino groups and supports the interpretation of the observed changes in the amine/amide region. Because this region contains overlapping contributions from amino, amide, and carboxylate groups, the peak-area analysis is considered semi-quantitative evidence of changes in the local chemical environment rather than a direct determination of the degree of amino-group protonation.

The C=O stretching shoulder near 1653 cm^−1^ (amide I), evident in neat chitosan, was attenuated in the films, while a new contribution near 1530 cm^−1^ became apparent, further indicating changes in the local environment of the chitosan chains. The chitosan C–O and C–O–C bands at approximately 1064 and 1024 cm^−1^ showed substantial changes in intensity and band profile, indicating reorganization of the chitosan–NaDES interaction network [49]. DES_GA2 itself contributed a characteristic carbonyl band near 1730 cm^−1^ (glycolic acid C=O stretching), whose profile differed in the films from that of pure DES_GA2, consistent with hydrogen bonding and acid–base interactions between glycolic acid and the chitosan matrix. This interpretation is consistent with previous studies showing that the acid used for chitosan solubilization affects intermolecular interactions and polymer organization, ultimately influencing film properties [50,51]. These spectral characteristics are qualitatively similar to those reported for the DES_GA3 system [42], but they provide a baseline for interpreting the effects of stoichiometry and the incorporation of botanical extracts in the following sections.

#### 3.1.2. Effect of Plant Extract Incorporation

**Figure 2:**
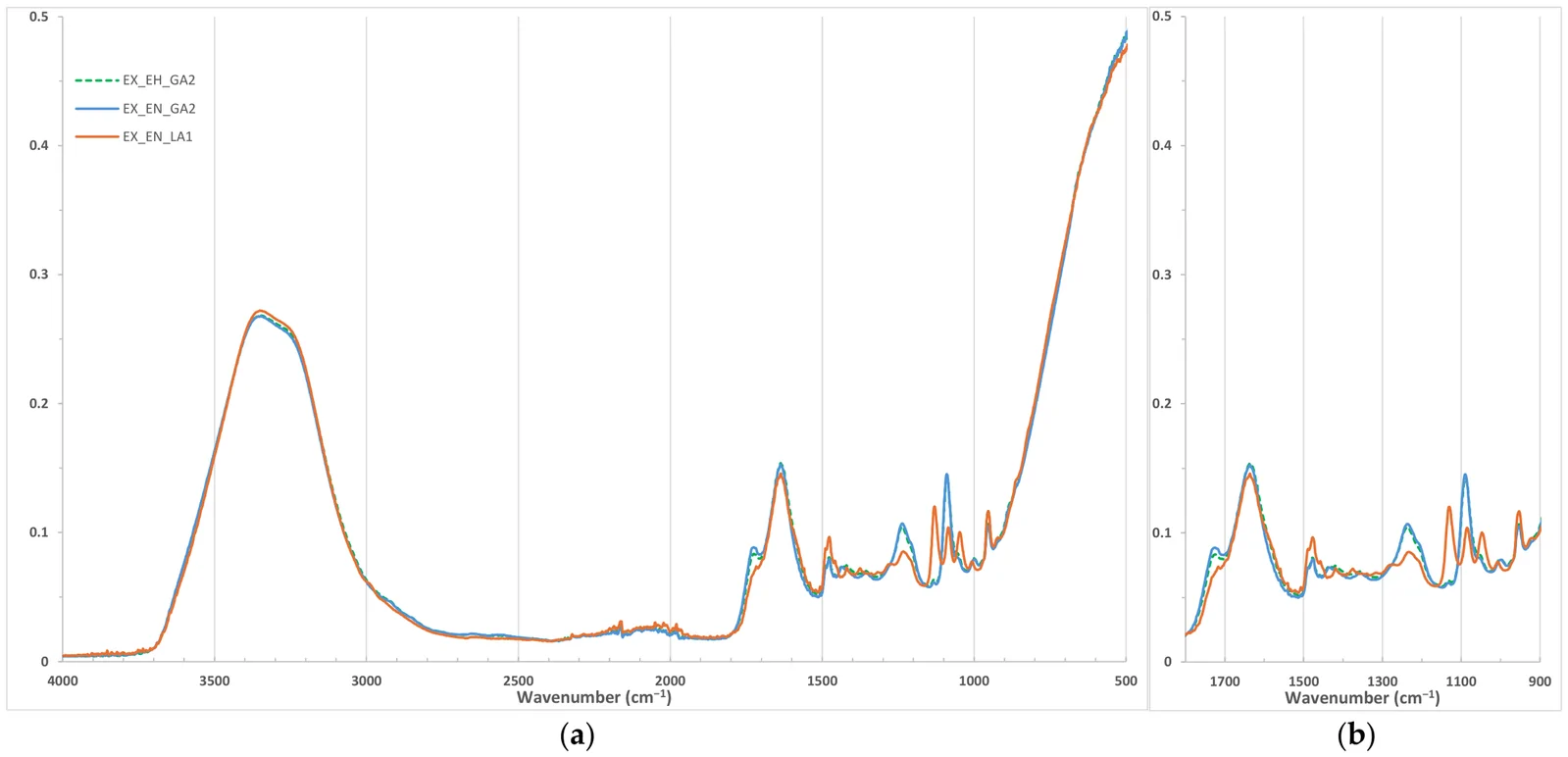
FTIR spectra of hawthorn and neem extracts obtained using 30% (*w*/*w*) aqueous DES_GA2 (EX_EH_GA2 and EX_EN_GA2) and DES_LA1 (EX_EN_LA1) as extraction media: (**a**) full spectral range; (**b**) enlarged 1800–900 cm^−1^ region.

As shown in Figure 2a, the spectra of the three NaDES-based extracts (Ex_EH_GA2, Ex_EN_GA2, Ex_EN_LA1) were qualitatively similar across the full spectral range, reflecting the dominant contribution of the aqueous NaDES medium. Spectral differentiation between extracts was more pronounced in the 1800–900 cm^−1^ region (Figure 2b).

**Figure 3:**
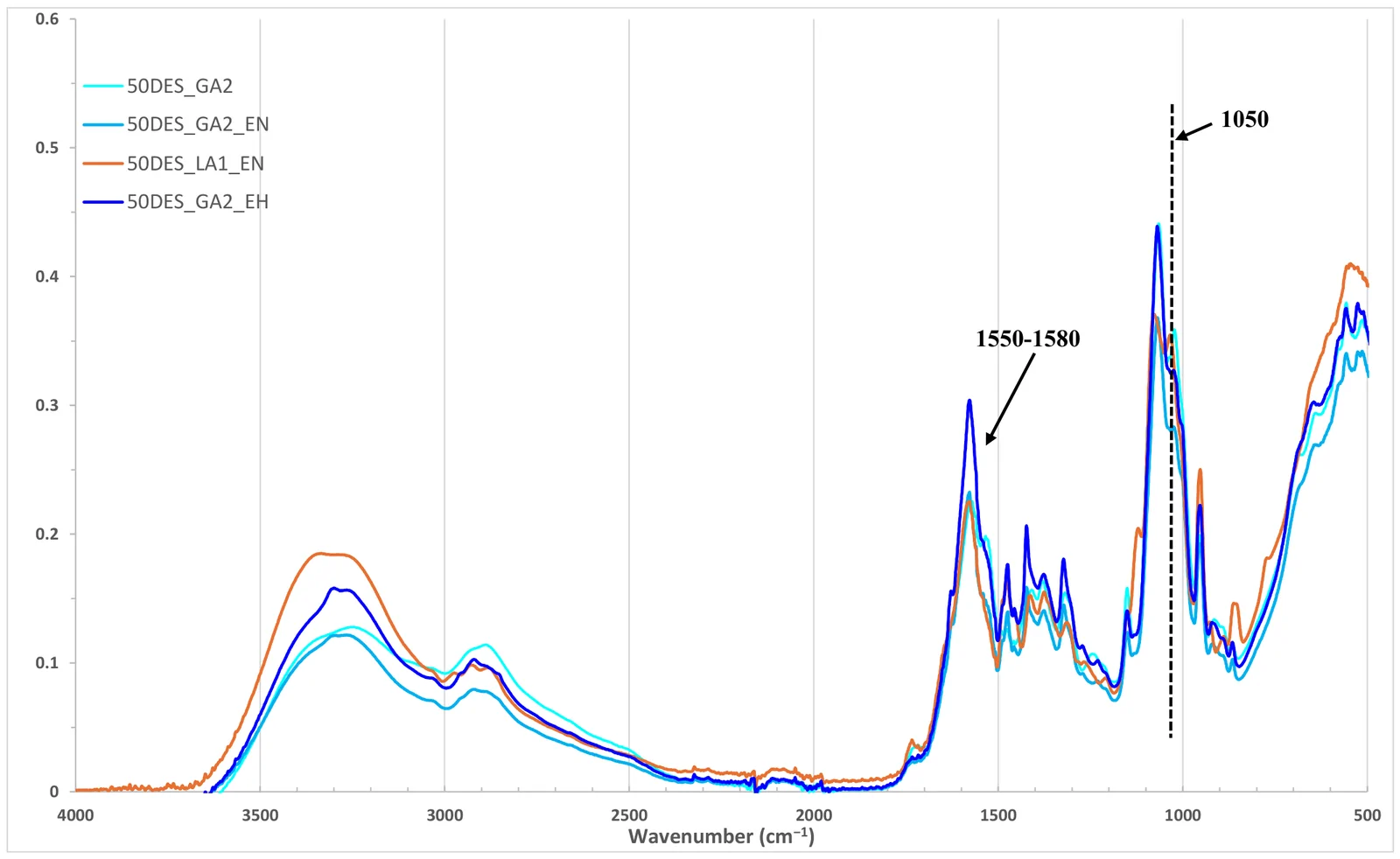
FTIR spectra of CS–NaDES films with and without plant extracts, including the comparative neem-containing DES_LA1 film.

As shown in Figure 3, incorporation of all extracts modified the FTIR spectrum of the 50DES_GA2 film while preserving its principal structural features. The most pronounced spectral changes were observed in the broad 3200–3500 cm^−1^ regions, together with a marked decrease in the band around 2930 cm^−1^. Changes were also observed in the 1550–1580 cm^−1^ range, associated with the chitosan amino environment, and near 1050 cm^−1^ in the fingerprint region, consistent with perturbation of the non-covalent interaction network by the extracted plant compounds. Differences between 50DES_GA2_EN and 50DES_LA1_EN, particularly in the O–H/N–H stretching region and the fingerprint region, further indicate that NaDES medium influences the molecular environment of the neem-containing films.

These findings are consistent with extract-induced spectral modifications reported for related chitosan–NaDES systems incorporating *Rosa roxburghii* and astaxanthin extracts [52,53]. Other researchers reported that some peaks were blue-shifted in the films that incorporated turmeric extracts [54].

### 3.2. Thermal Properties

**Figure 4:**
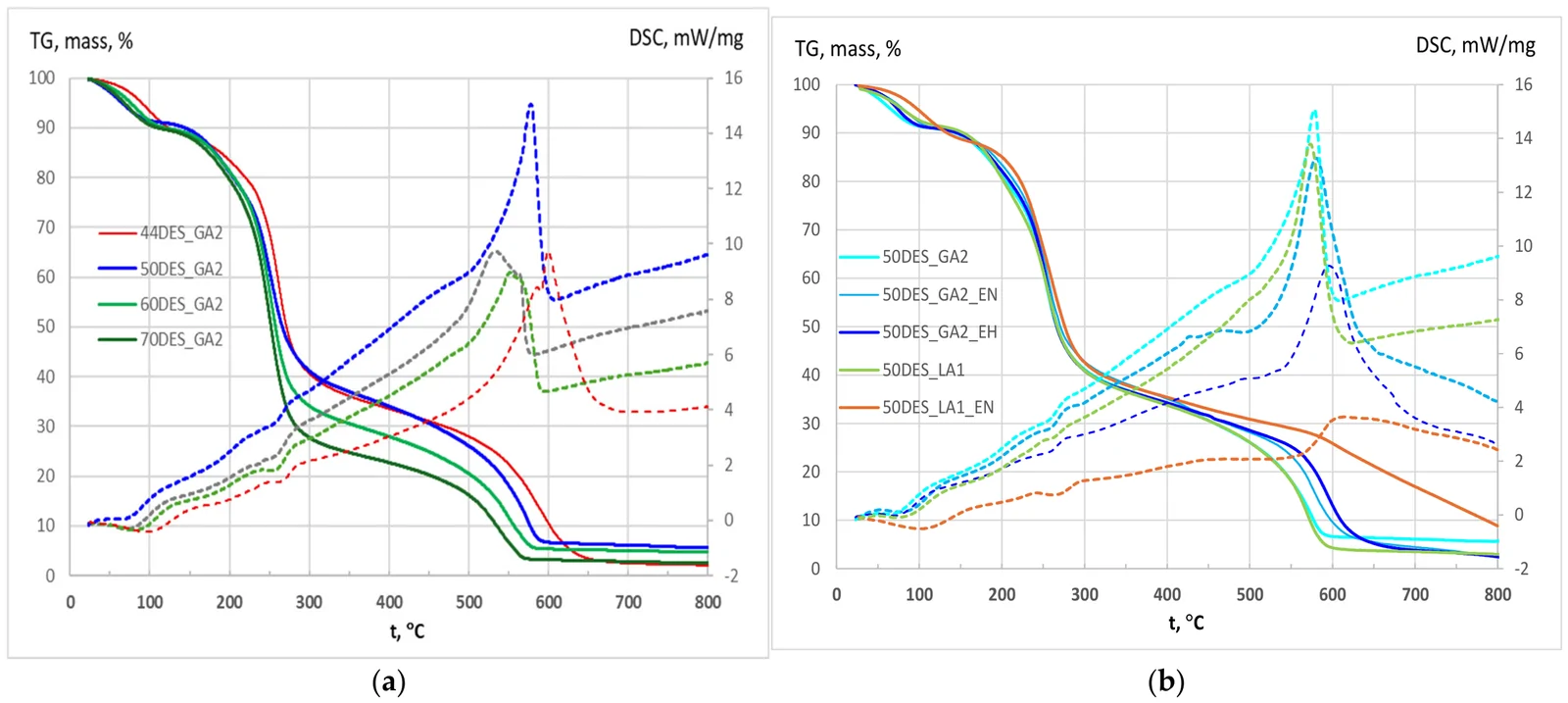
TG (solid lines) and DSC (dashed lines) curves of chitosan films: (**a**) effect of DES_GA2 content; (**b**) effect of plant extract incorporation and comparison between DES_GA2- and DES_LA1-based formulations. The TG–DSC curves for the extract-free 50DES_LA1 film were previously reported in Ref. [42] and are replotted here for comparative purposes.

As shown in Figure 4, TG–DSC analysis revealed a common multistep thermo-oxidative degradation pattern across all formulations: an initial mass loss below 140 °C, a main degradation stage between approximately 140 and 300 °C associated with an endothermic event centered at 253–263 °C, and high-temperature oxidative degradation above 300 °C, accompanied by a pronounced exothermic combustion event. Because the measurements were performed in air, the observed thermal events may involve contributions from both thermal decomposition and oxidative processes, particularly at higher temperatures.

The initial mass loss (9–12%) below 140 °C (Table 1) is primarily attributable to desorption of free and bound water, evidenced by a weak endothermic event with minima between 64 and 101 °C. In the principal degradation stage (140–300 °C), the mass loss increased systematically with DES_GA2 content (from 48.7% for 44DES_GA2 to 61.5% for 70DES_GA2) reflecting the increasing thermal contribution of NaDES-rich domains.

Concurrently, the onset of this stage shifted to lower temperatures with increasing DES_GA2 loading (Figure 4a), indicating that NaDES incorporation reduces the onset temperature of the main degradation step, consistent with the plasticizing-induced increase in chain mobility. The maximum of the high-temperature oxidative exothermic effect also decreased progressively with DES_GA2 content (from 599.9 °C for 44DES_GA2 to 534.3 °C for 70DES_GA2), suggesting that NaDES content modulates the structure of the residual carbonaceous phase and its susceptibility to oxidation.

Extract incorporation shifted the first endothermic minimum upward (from 64.0 °C for 50DES_GA2 to 76.2 and 70.2 °C for 50DES_GA2_EN and 50DES_GA2_EH, respectively), indicating that plant compounds modify water binding within the matrix. For the DES_LA1-based system, neem incorporation produced more pronounced changes in the high-temperature oxidative stage. The 300–600 °C mass loss decreased from 36.6 to 26.6%, and the exothermic maximum shifted from 573.3 to 613.8 °C, while the corresponding change in the DES_GA2 matrix was negligible (578.3 to 580.1 °C). This NaDES-dependent response indicates that the influence of botanical extract on the high-temperature thermo-oxidative degradation behavior depends on the NaDES formulation. A distinction between intrinsic thermal decomposition and oxidative degradation would require complementary measurements under an inert atmosphere and is therefore beyond the scope of the present study. As TG–DSC was primarily used to assess mass loss and degradation behavior, future DMA measurements would provide a more detailed evaluation of the glass transition and thermomechanical behavior of these films.

### 3.3. UV–Vis Absorption and Photoluminescence Properties

**Figure 5:**
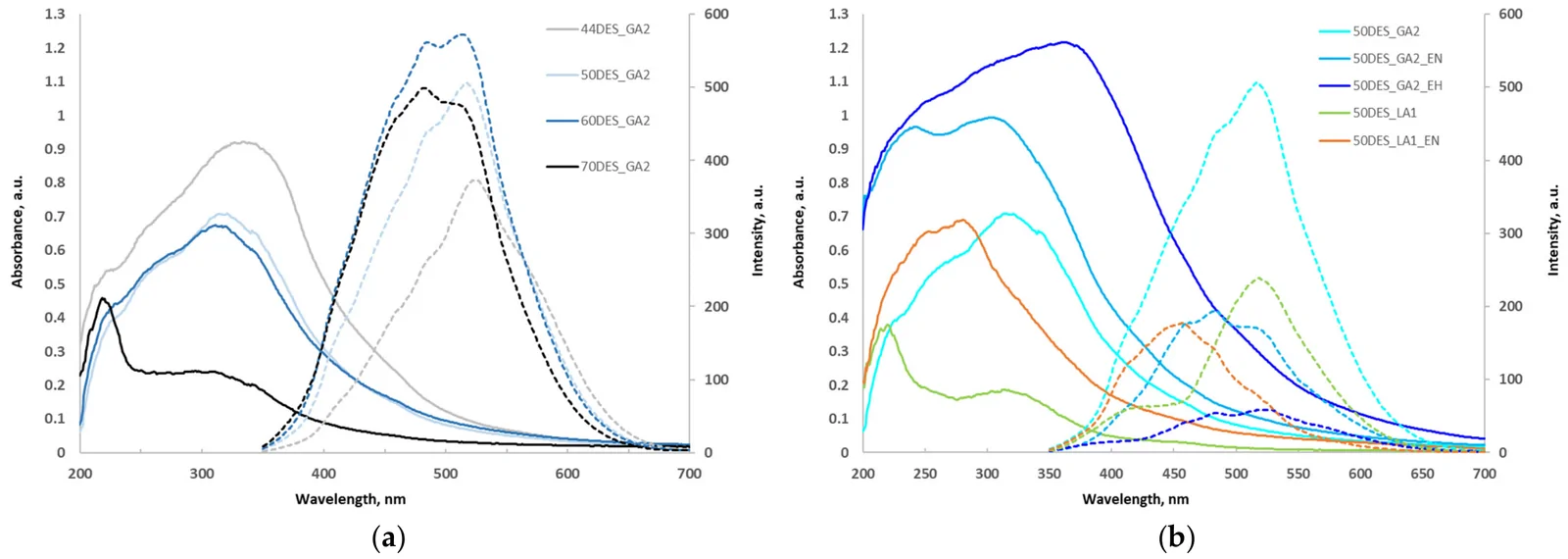
UV–Vis absorption (solid lines) and photoluminescence (PL, dashed lines) spectra of chitosan films: (**a**) effect of DES_GA2 content; (**b**) effect of hawthorn and neem extracts in DES_GA2-based films and comparison of extract-free and neem-containing films prepared with DES_GA2 and DES_LA1.

As shown in Figure 5a, CS–DES_GA2 films exhibited pronounced UV absorption between 220 and 350 nm together with broad visible photoluminescence. Films containing 44–60 wt% DES_GA2 showed a broad absorption band centered near 310–320 nm, with the highest absorbance for 44DES_GA2. The 70DES_GA2 film presented a qualitatively different profile: a strong absorption maximum near 220 nm and a lower-intensity broad shoulder extending to approximately 350 nm. A similar absorption feature in the UVC region, at approximately 235 nm, has previously been observed in chitosan-based materials [55].

Photoluminescence emission shifted progressively from the blue towards the green spectral region as DES_GA2 content decreased, and the emission intensity showed a non-monotonic dependence on NaDES content, with the strongest response at intermediate compositions. This behavior is consistent with nonconventional luminescence arising from the clustering of electron-rich groups and restricted intramolecular motion in the condensed state, a mechanism increasingly described for polysaccharide systems in terms of aggregation-induced emission (AIE) [56,57]. However, the present steady-state spectroscopic data do not provide direct evidence of the underlying emission mechanism, and the AIE interpretation should therefore be regarded as a plausible explanation of the observed spectral behavior.

Extract incorporation substantially broadened UV absorption toward the UVA region, as shown in Figure 5b, with the hawthorn-containing film (50DES_GA2_EH) showing the most pronounced extension into the visible range. By contrast, the neem-containing film (50DES_GA2_EN) exhibited relatively stronger absorption in the UVB region. Both botanical extracts therefore enhanced the UV-screening capacity of the films, supporting their potential as UV-protective active packaging materials. Neem incorporation caused a marked decrease in PL intensity in both NaDES systems, while hawthorn incorporation similarly reduced PL intensity in the DES_GA2 matrix. This apparent PL quenching may arise, at least in part, from an inner-filter effect associated with the enhanced UV–Vis absorption of the extract-containing films, which can attenuate the excitation and/or emitted light. Interactions between extract constituents and the chitosan–NaDES matrix may additionally modify the emissive states, as the molecules become dispersed among chitosan chains and disturb the aggregation state that was responsible for AIE. However, the available steady-state UV–Vis and PL data do not allow these contributions to be unequivocally distinguished.

The magnitude and spectral profile of the PL decrease varied across systems, indicating that both the botanical source and the NaDES medium influence the observed photophysical response.

### 3.4. Physical Appearance, Morphology, and Mechanical Properties

**Figure 6:**
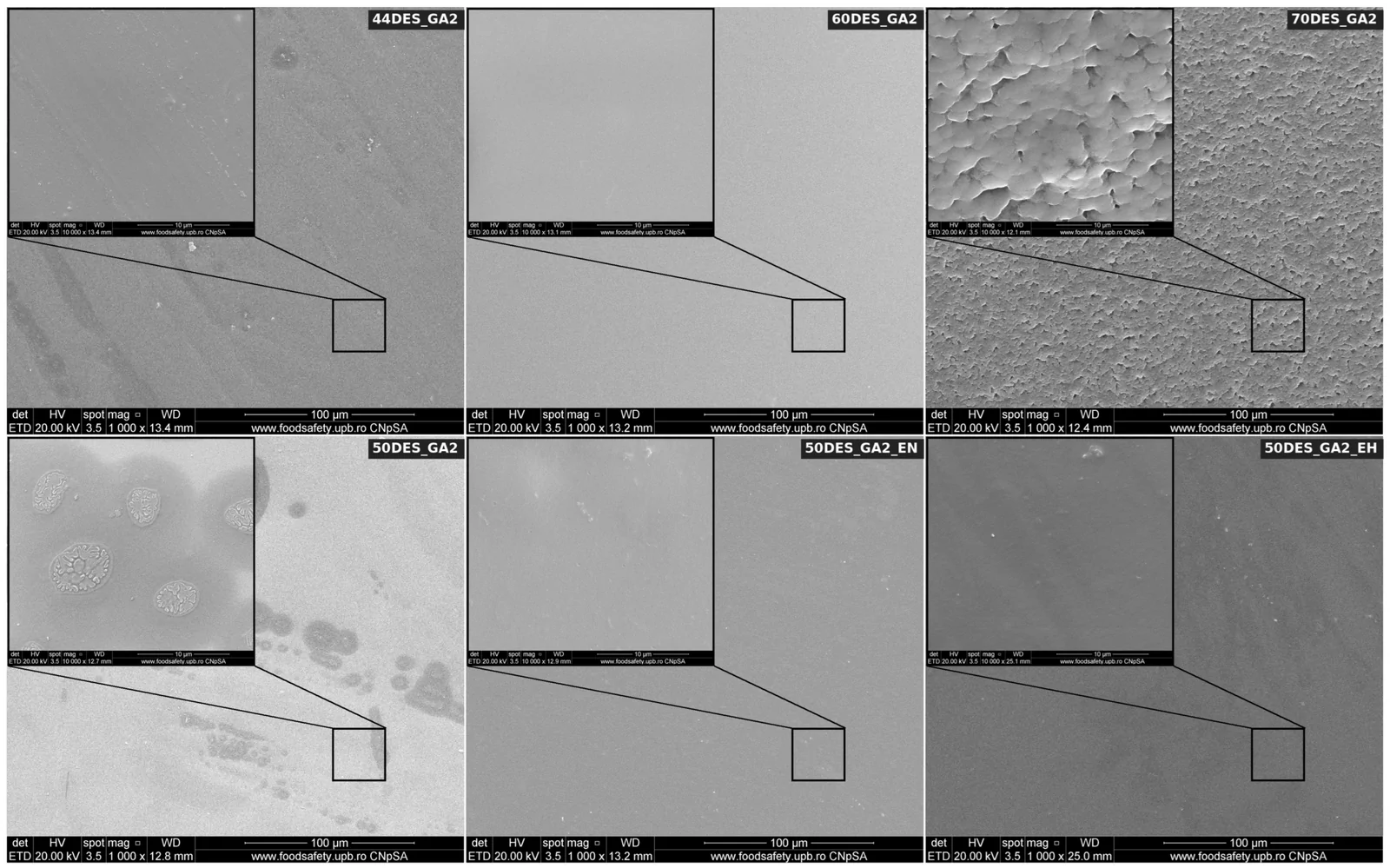
SEM images of the surfaces of CS–NaDES films acquired at magnifications of 1000× and 10,000×.

All films were macroscopically homogeneous and visually transparent. Extract-free films ranged from colorless to faintly opalescent, whereas extract incorporation imparted a light-brown coloration while preserving visual transparency (Figure S4). As shown in Figure 6, SEM images (1000× and 10,000×) revealed continuous surfaces without evident macroscopic defects across all formulations, with surface texture varying with DES_GA2 content.

Lower NaDES contents yielded comparatively smooth surfaces, while the surface of 70DES_GA2 contained numerous approximately spherical structures, 1.8–2.5 μm in diameter. A progressive increase in surface heterogeneity with increasing NaDES content has also been reported in related CS–NaDES systems [20,58,59]. Localized irregularities were observed for 50DES_GA2; extract-loaded films (50DES_GA2_EN and 50DES_GA2_EH) displayed comparatively uniform and smooth surfaces.

Film thickness (Table 2) increased from 43.6 ± 1.8 μm (44DES_GA2) to 66.8 ± 3.3 μm (70DES_GA2), without a significant difference between the 50 and 60 wt% formulations (*p* > 0.05).

Moisture content (MC) followed a monotonic increase from 18.4% to 29.8% across the same DES_GA2 range, consistent with the hygroscopic nature of the NaDES components. Water solubility (WS) was statistically similar for 44DES_GA2 and 50DES_GA2 (41.2 and 40.6%, respectively) but increased significantly at 60 and 70 wt% DES_GA2, reaching 55.0% at the highest loading.

**Figure 7:**
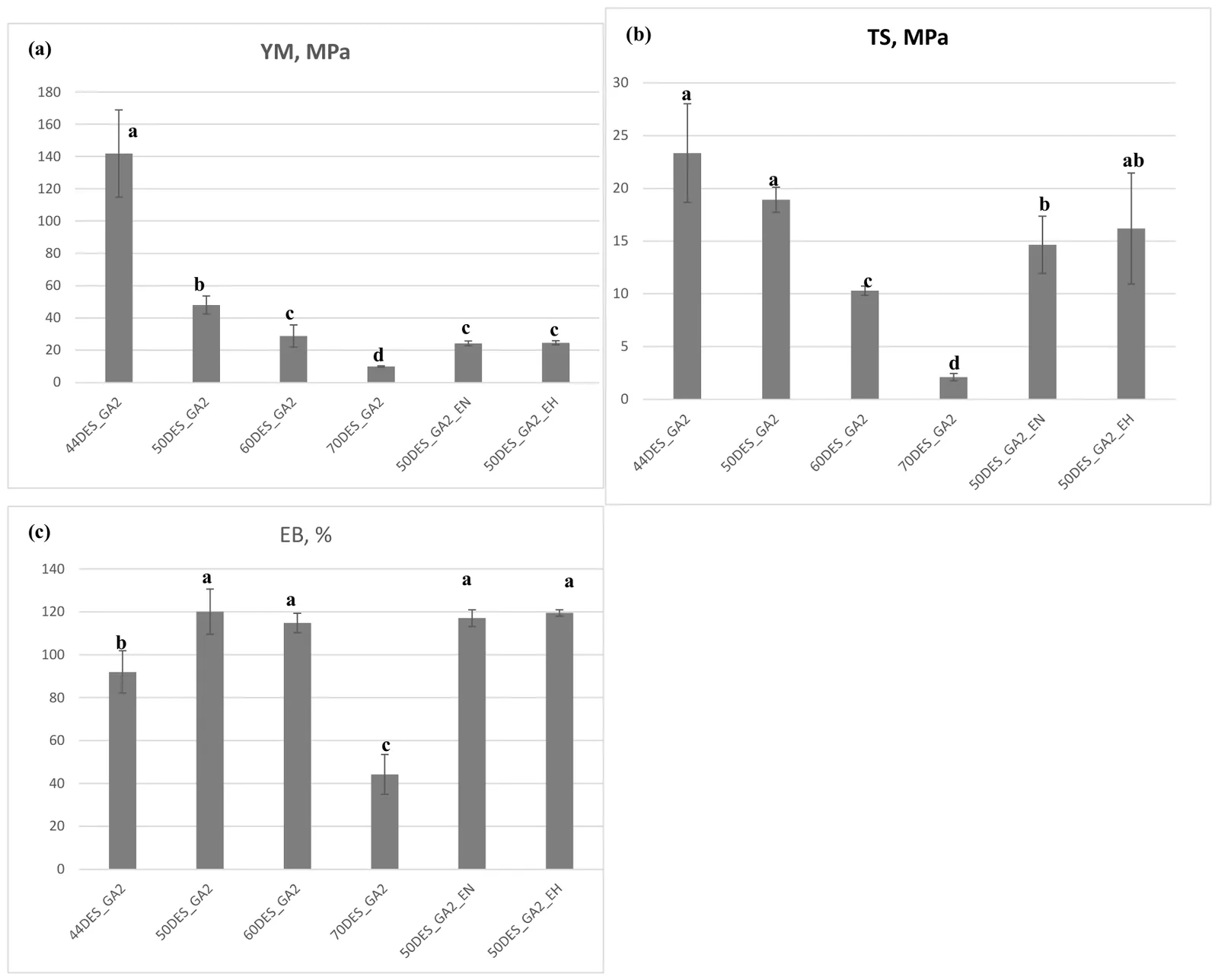
Effect of NaDES content and plant extract incorporation on the mechanical properties of CS–NaDES films: (**a**) Young’s modulus (YM); (**b**) tensile strength (TS); and (**c**) elongation at break (EB). Different letters indicate statistically significant differences among formulations (*p* < 0.05).

Representative stress–strain curves are shown in Figure S5, while the corresponding Young’s modulus, tensile strength, and elongation at break values are summarized in Figure 7. Young’s modulus decreased sharply with increasing DES_GA2 content, from approximately 140 MPa for 44DES_GA2 to approximately 10 MPa for 70DES_GA2, confirming progressive plasticization of the chitosan matrix (Figure 7a). Tensile strength followed the same trend, falling below 5 MPa for 70DES_GA2 while remaining above 20 MPa for formulations with lower NaDES content, as is shown in Figure 7b. Elongation at break (EB) was approximately 120% for both 50DES_GA2 and 60DES_GA2 but decreased to approximately 44% for 70DES_GA2 (Figure 7c), indicating that the balance between chain mobility and network integrity is lost at the highest DES_GA2 loadings.

Extract incorporation significantly decreased YM and TS, with reductions of 49.4% and 22.6%, respectively, for the 50DES_GA2_EN formulation compared with the film without extract, suggesting that the incorporated plant-derived compounds further disrupted the chitosan network. In contrast, EB remained statistically unchanged (*p* > 0.05). Among the investigated formulations containing DES_GA2 (44–70 wt%), the 50DES_GA2 formulation exhibited the most favorable balance of mechanical, hydration, and morphological properties and was, therefore, selected as the base formulation for the incorporation of the extract. A pure chitosan film (0 wt% DES_GA2) was not included as a baseline; thus, this assessment is restricted to the investigated CS–NaDES composition range.

### 3.5. Water Vapor Permeability

**Figure 8:**
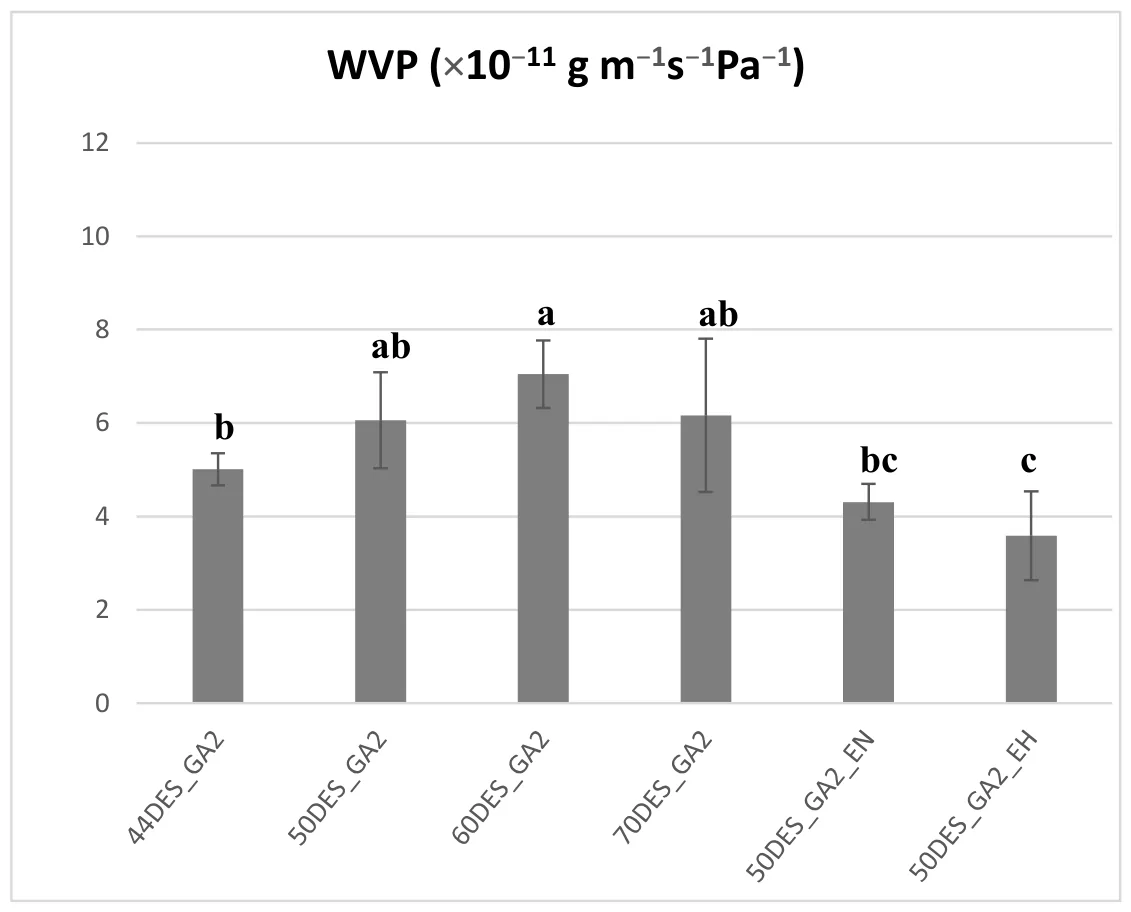
Water vapor permeability (WVP) of CS–NaDES films as a function of DES_GA2 content and plant extract incorporation. Different letters indicate statistically significant differences among formulations (*p* < 0.05).

As shown in Figure 8, WVP values ranged from approximately 3.5 to 7.0 × 10^−11^ g m^−1^ s^−1^ Pa^−1^ across the investigated films, within the range reported for comparable CS-based packaging materials [21,52,60,61]. Among extract-free DES_GA2 films, WVP increased from 44DES_GA2 to a maximum at 60DES_GA2 and then decreased for 70DES_GA2, deviating from a simple monotonic relationship with NaDES content. The non-monotonic trend indicates that water vapor transport is governed not only by matrix hydrophilicity but also by changes in film thickness, polymer chain packing density, and matrix organization at high NaDES contents.

Extract incorporation into 50DES_GA2 reduced WVP to approximately 4.2 × 10^−11^ g m^−1^ s^−1^ Pa^−1^ (50DES_GA2_EN) and 3.5 × 10^−11^ g m^−1^ s^−1^ Pa^−1^ (50DES_GA2_EH). The reduction was statistically significant for the hawthorn-containing film (*p* < 0.05) but not for the neem-containing film (*p* > 0.05). The comparatively smooth and homogeneous surfaces observed by SEM for the extract-loaded films are consistent with a more compact matrix organization that may partly account for the lower permeability.

To further assess the effect of ChCl:GA stoichiometry, the mechanical, hydration, and water vapor barrier properties of the present DES_GA2 films were compared with those previously reported for the corresponding DES_GA3 formulations (Table 3). At equivalent NaDES contents, the two systems exhibited distinct property profiles, indicating that the ChCl:GA molar ratio represents an additional formulation variable beyond the total NaDES content.

At equivalent NaDES contents, DES_GA2 films consistently exhibited higher MC and WS than the corresponding DES_GA3 films. A particularly pronounced difference was observed for WVP: DES_GA2 films showed values of 5.0–7.1 × 10^−11^ g m^−1^ s^−1^ Pa^−1^, compared with 2.9–4.6 × 10^−11^ g m^−1^ s^−1^ Pa^−1^ for DES_GA3 films. Moreover, whereas WVP generally decreased with increasing DES_GA3 content, the DES_GA2 series showed a non-monotonic dependence, reaching a maximum at 60 wt% NaDES. These differences indicate that increasing the ChCl:GA molar ratio from 1:3 to 1:2 modifies water affinity and water-vapor transport within the CS–NaDES matrix.

The mechanical response was also dependent on ChCl:GA stoichiometry, although the differences were more composition-dependent. DES_GA2 generally exhibited higher extensibility at low and intermediate NaDES contents, most notably at 50 wt%, where EB increased from 90.5% for DES_GA3 to 120.1% for DES_GA2, while YM decreased from 131.0 to 48.0 MPa. At higher NaDES contents, however, the differences between the two systems became less systematic. Thus, the effect of ChCl:GA stoichiometry cannot be described solely as a change in plasticization intensity but rather reflects a modification of the balance between chain mobility, water affinity, and barrier performance.

Despite these differences, the 50 wt% NaDES formulation provided a favorable overall balance of mechanical and barrier properties in both systems, suggesting a robust formulation window for ChCl:GA-based CS films.

To determine whether the stoichiometry-dependent differences observed for the extract-free films were maintained after plant extract incorporation, the properties of the hawthorn-containing formulations prepared with ChCl:GA molar ratios of 1:2 and 1:3 were further compared (Table 4).

The comparison shows that the higher extensibility observed for the 1:2 system was also evident after hawthorn incorporation. EB was 119.5% for 50DES_GA2_EH compared with 79.1% for 50DES_GA3_EH, corresponding to an approximately 51% higher value, while YM was lower (24.6 vs. 29.9 MPa). MC and WS were also higher for the DES_GA2 film, consistent with the greater water affinity observed for the corresponding extract-free system. In contrast to the trend observed for the extract-free films, WVP was numerically lower for the hawthorn-containing DES_GA2 film than for its DES_GA3 counterpart (3.6 vs. 4.1 × 10^−11^ g m^−1^ s^−1^ Pa^−1^). Thus, although the enhanced extensibility and greater water affinity associated with the 1:2 stoichiometry persisted after hawthorn incorporation, the barrier response differed from that of the extract-free films, indicating that the final property profile depends on the interplay between NaDES stoichiometry and plant extract incorporation.

### 3.6. Total Phenolic Content and Antioxidant Activity

Preliminary screening using aqueous DES_LA1 showed that TPC increased markedly with NaDES concentration, as is shown in Figure S6. Aqueous NaDES systems covering a broad range of NaDES contents have been employed for the extraction of bioactive compounds from different plant matrices, with water addition generally reducing solvent viscosity and facilitating mass transfer [22,24,25,62]. In the present study, 30% (*w*/*w*) DES_LA1 already provided a high TPC, statistically comparable to that obtained with 50% aqueous ethanol. This concentration was therefore selected for the subsequent extraction experiments as a practical compromise between extraction efficiency and NaDES consumption.

**Figure 9:**
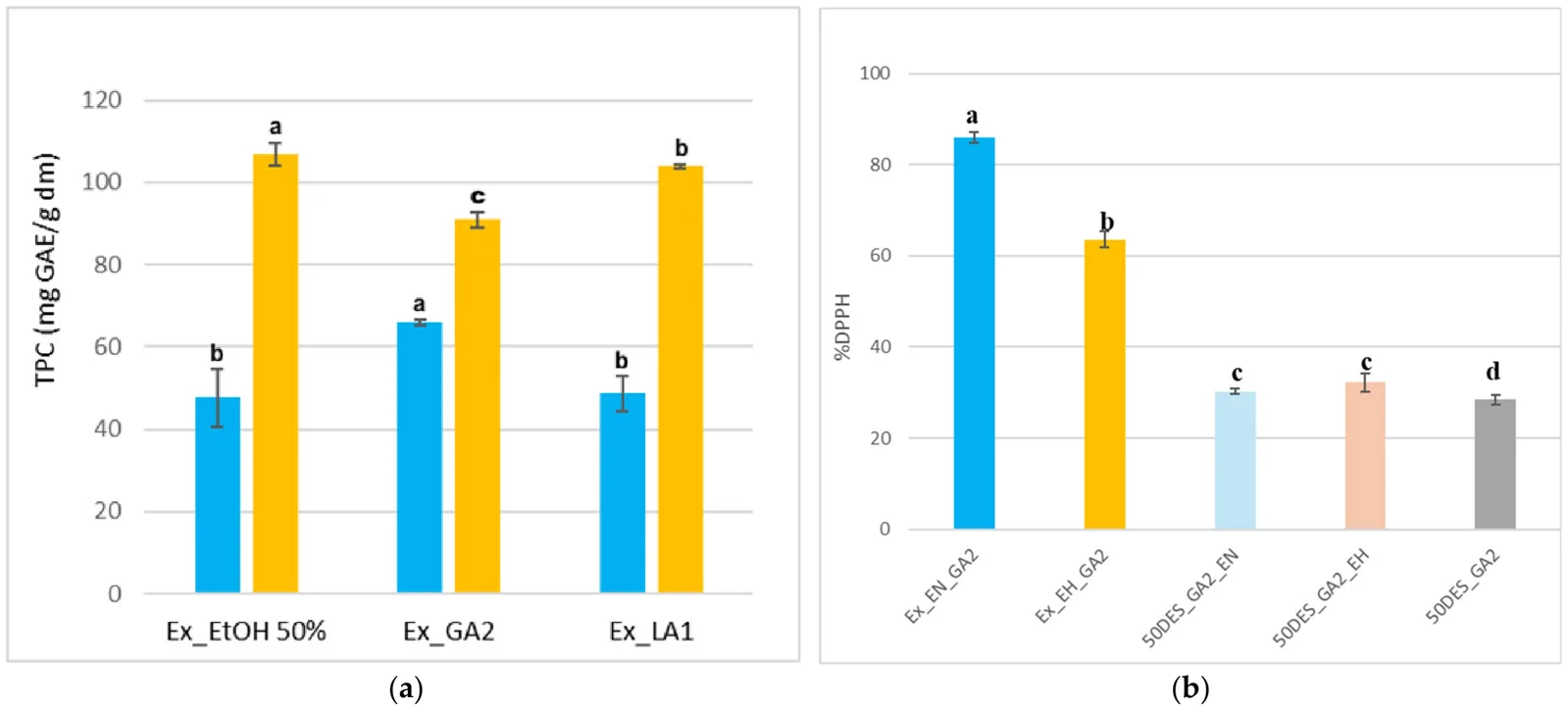
(**a**) Total phenolic content (TPC) of neem (blue) and hawthorn (orange) extracts obtained using different extraction media; (**b**) DPPH radical scavenging activity of DES_GA2-based neem and hawthorn extracts and the corresponding CS–NaDES films. Different letters indicate statistically significant differences among samples (*p* < 0.05).

NaDES-assisted extraction performance differed between the two plant materials, as can be observed in Figure 9a. DES_GA2 increased neem TPC by approximately 39% relative to 50% aqueous ethanol, yielding approximately 66 mg GAE g^−1^ DM. In contrast, hawthorn TPC in DES_GA2 (approximately 91–92 mg GAE g^−1^ DM) was approximately 14–15% lower than the ethanolic reference, although DES_LA1 recovered values close to the ethanol standard for hawthorn. These divergent extraction profiles demonstrate that NaDES efficiency is plant-matrix-dependent and cannot be generalized across botanical species.

As shown in Figure 9b, the DPPH radical scavenging activity did not follow the trend observed for TPC. Thus, neem extract exhibited scavenging activity exceeding 85%, substantially higher than hawthorn extract (approximately 63–64%), despite its lower phenolic content. This decoupling between TPC and radical scavenging capacity indicates that the antioxidant potency of polyphenol extracts is determined by the specific chemical identity and stoichiometry of the active compounds rather than by their total mass. After incorporation into the CS–DES_GA2 matrix, both extracts retained measurable radical scavenging activity (*p* < 0.05 vs. extract-free 50DES_GA2), although film activity was substantially lower than that of the free extracts, consistent with restricted accessibility of embedded antioxidant compounds [63]. From a practical perspective, these findings indicate that TPC alone may not be sufficient for selecting plant sources for active packaging applications. Therefore, screening based on total phenolic content should be complemented by direct evaluation of radical scavenging activity to better assess the antioxidant potential of plant extracts.

### 3.7. Antibacterial Properties of Chitosan Films

Inhibition zone measurements are summarized in Table 5, and representative images of the antibacterial assays are shown in Figure S7. The extract-free 50DES_GA2 film exhibited negligible inhibitory activity against both tested organisms. Both plant extracts showed measurable antibacterial activity against both tested organisms, with Ex_EH_GA2 producing larger inhibition zones (9.66 ± 0.57 mm against *E. coli*; 4.66 ± 0.57 mm against *B. Subtilis*) than Ex_EN_GA2 (4.66 ± 0.57 mm against *E. coli*; 3.00 ± 0.99 mm against *B. Subtilis*). This differential response to Gram-positive and Gram-negative strains is consistent with observations in related chitosan-based systems [64].

After incorporation into the CS matrix, antibacterial activity against *E. coli* was retained for both extract-loaded films (50DES_GA2_EN: 2.33 ± 0.33 mm; 50DES_GA2_EH: 1.33 ± 0.33 mm), while no inhibition against *B. subtilis* was detectable. Notably, the neem-containing film showed a larger *E. coli* inhibition zone than the one containing hawthorn, despite the lower activity of the free neem extract. This reversal may reflect differences in extract–matrix interactions and in the availability and diffusion of active compounds from the two films. The absence of detectable activity against *B. subtilis* after extract incorporation may reflect differences in bacterial susceptibility to the bioactive compounds released from the CS–NaDES matrix.

## 4. Conclusions

This study demonstrates that ChCl:GA stoichiometry is an important compositional parameter governing the structure–property relationships of CS–NaDES films, most likely through modifications to the network of intermolecular interactions established between chitosan and the NaDES components. Compared with the previously investigated ChCl:GA 1:3 system, decreasing the relative glycolic acid content to a 1:2 molar ratio produced a distinct mechanical, hydration, and barrier profile. At 50 wt% NaDES, the 1:2 formulation showed markedly enhanced flexibility, with elongation at break increasing from 90.5% to 120.1% and Young’s modulus decreasing from 131.0 to 48.0 MPa relative to the 1:3 system. However, DES_GA2 films also exhibited systematically higher water-related and barrier properties, indicating that the effect of stoichiometry involves a trade-off between plasticization and water barrier performance rather than a uniform improvement in film properties. Within the NaDES content range investigated (44–70 wt%), 50 wt% provided a favorable overall balance of mechanical and barrier properties in both ChCl:GA systems, supporting its selection as the base formulation for further functionalization.

Plant extract incorporation introduced additional composition-dependent changes in film properties and functionality. Neem showed higher DPPH radical scavenging activity than hawthorn despite its lower TPC content, demonstrating that antioxidant performance cannot be inferred from TPC alone. This finding has practical relevance for the development of active packaging: selection of plant raw materials or extracts should not rely solely on TPC as a screening parameter but should also include direct evaluation of free radical scavenging activity to identify extracts with the most suitable antioxidant functionality. Both extracts enhanced UV shielding and retained antioxidant and antibacterial functionality after incorporation into the films. Hawthorn produced the most pronounced improvement in water vapor barrier performance, reducing WVP by approximately 41% relative to the extract-free 50DES_GA2 film, while both extract-containing films showed measurable antibacterial activity against *E. coli*.

The comparison of hawthorn-containing films further showed that the effect of ChCl:GA stoichiometry persisted after plant extract incorporation. Increasing the ChCl:GA molar ratio from 1:3 to 1:2 increased elongation at break from 79.1% to 119.5%, while the higher moisture content and water solubility characteristic of DES_GA2 were maintained. In contrast to the extract-free films, however, the hawthorn-containing DES_GA2 film exhibited a slightly lower WVP than its DES_GA3 counterpart (3.6 vs. 4.1 × 10^−11^ g m^−1^ s^−1^ Pa^−1^), indicating that the barrier response is influenced by the interplay between NaDES stoichiometry and plant extract incorporation. Finally, comparison of neem-loaded films based on DES_GA2 and DES_LA1 showed that the response to the same botanical source also depends on NaDES composition, as evidenced by differences in molecular environment, optical response, and high-temperature oxidative behavior.

Together, these findings show that both NaDES composition, including HBD identity and HBA:HBD stoichiometry, and the choice of botanical extract are important factors in determining the properties and functionality of CS–NaDES active packaging films. The dual use of NaDES as a plasticizer and extraction medium therefore provides a versatile strategy for developing active chitosan-based packaging materials. Further studies should investigate controlled release kinetics, long-term stability, and film performance in real food systems under relevant storage conditions, including antimicrobial evaluation against a broader range of foodborne and spoilage microorganisms selected according to the specific food matrix. Food-contact safety and overall and specific migration testing should also be addressed to establish the suitability of the developed films for food packaging applications.

## Figures and Tables

**Table 1:** Principal thermal analysis parameters for the CS–NaDES films.

Sample	Mass Loss (%) RT–140 °C	Endo I (°C)	Endo II (°C)	Mass Loss (%) 140–300 °C	Mass Loss (%) 300–600 °C	Exo (°C)
44DES_GA2	10.6	95.8	258.9	48.7	39.0	599.9
50DES_GA2	9.3	64.0	255.0	49.5	36.4	578.3
60DES_GA2	10.4	80.5	253.1	55.5	30.0	552.3
70DES_GA2	10.7	70.9	257.0	61.5	25.9	534.3
50DES_GA2_EN	11.7	76.2	259.0	53.1	34.7	580.1
50DES_GA2_EH	12.3	70.2	256.0	53.4	33.5	594.8
50DES_LA1 *	9.1	83.6	263.0	50.0	36.6	573.3
50DES_LA1_EN	12.1	100.8	261.4	52.4	26.6	613.8

* Thermal data for 50DES_LA1 previously reported [42]; mass loss values recalculated from original TG data using the temperature intervals applied here.

**Table 2:** Thickness, moisture content, and water solubility of the CS–NaDES films.

Sample	d (μm)	MC (%)	WS (%)
44DES_GA2	43.6 ± 1.8 ^c^	18.4 ± 0.3 ^e^	41.2 ± 2.7 ^c^
50DES_GA2	51.2 ± 2.2 ^b^	21.4 ± 0.5 ^d^	40.6 ± 3.1 ^c^
60DES_GA2	52.6 ± 5.8 ^b^	25.3 ± 0.6 ^b^	47.8 ± 1.7 ^b^
70DES_GA2	66.8 ± 3.3 ^a^	29.8 ± 1.4 ^a^	55.0 ± 1.9 ^a^
50DES_GA2_EN	45.0 ± 4.7 ^bc^	21.5 ± 0.5 ^d^	41.2 ± 1.6 ^c^
50DES_GA2_EH	48.4 ± 5.7 ^bc^	22.8 ± 0.1 ^c^	39.2 ± 1.5 ^c^

Different superscript letters indicate statistically significant differences (*p* < 0.05).

**Table 3:** Comparison of mechanical, hydration, and water vapor barrier properties of CS–NaDES films prepared with ChCl:GA molar ratios of 1:2 and 1:3.

NaDES Content (wt%)	ChCl:GA	YM (MPa)	TS (MPa)	EB (%)	MC (%)	WS (%)	WVP (×10^−11^ g m^−1^ s^−1^ Pa^−1^)
44	1:2	141.8± 27.0	23.3 ± 4.7	92.0 ± 9.9	18.4 ± 0.3	41.2 ± 2.7	5.0 ± 0.4
	1:3 *	175.1± 17.0	21.2 ± 8.8	45.0 ± 18.3	17.5 ± 0.2	31.9 ± 1.3	4.6 ± 0.5
50	1:2	48.0± 5.5	18.9 ± 1.2	120.1 ± 10.6	21.4 ± 0.5	40.6 ± 3.1	6.1 ± 1.0
	1:3 *	131.0± 7.8	23.9 ± 1.2	90.5 ± 6.1	19.5 ± 0.3	34.8 ± 0.4	4.3 ± 0.3
60	1:2	28.8± 6.9	10.3 ± 0.4	114.8 ± 4.5	25.3 ± 0.6	47.8 ± 1.7	7.1 ± 0.7
	1:3 *	14.8± 3.9	9.3 ± 1.3	98.3 ± 8.5	21.2 ± 0.7	43.3 ± 0.5	3.2 ± 0.6
70	1:2	10.0± 0.4	2.1 ± 0.4	44.8± 9.3	29.8 ± 1.4	55.0 ± 1.9	6.2 ± 1.6
	1:3 *	11.7± 1.0	4.7 ± 1.3	63.2 ± 11.7	24.7 ± 1.3	50.5 ± 1.0	2.9 ± 0.3

* Data for the ChCl:GA 1:3 system were previously reported in [42]. Because the two datasets were obtained in separate experimental studies, no direct statistical comparison was performed between the DES_GA2 and DES_GA3 series.

**Table 4:** Comparison of the mechanical, hydration, and water vapor barrier properties of hawthorn-containing CS–NaDES films prepared with ChCl:GA molar ratios of 1:2 and 1:3 at 50 wt% NaDES.

Film	ChCl:GA	YM (MPa)	TS (MPa)	EB (%)	MC (%)	WS (%)	WVP (×10^−11^ g m^−1^ s^−1^ Pa^−1^)
50DES_GA2_EH	1:2	24.6± 1.3	16.2 ± 5.3	119.5± 1.5	22.8 ± 0.1	39.2 ± 1.5	3.6 ± 1.0
50DES_GA3_EH	1:3 *	29.9± 1.8	13.0 ± 3.3	79.1 ± 1.5	20.0 ± 1.2	35.7 ± 1.3	4.1 ± 1.4

* Data for 50DES_GA3_EH were previously reported in [42]. Because the two datasets were obtained in separate experimental studies, no direct statistical comparison was performed.

**Table 5:** Antibacterial activity of extracts and extract-containing films, expressed as inhibition zone (IZ, mm).

Sample	IZ *E. coli* (−)	IZ *B. subtilis* (+)
50DES_GA2	0.15 ± 0.03 ^d^	0.12 ± 0.17 ^c^
Ex_EN_GA2	4.66 ± 0.57 ^b^	3.00 ± 0.99 ^b^
50DES_GA2_EN	2.33 ± 0.33 ^bc^	nd
Ex_EH_GA2	9.66 ± 0.57 ^a^	4.66 ± 0.57 ^a^
50DES_GA2_EH	1.33 ± 0.33 ^c^	nd

nd = not detected. Different lowercase letters indicate statistically significant differences (*p* < 0.05).

## Data Availability

The original contributions presented in this study are included in the article/Supplementary Materials. Further inquiries can be directed to the corresponding author.

## Supplementary Materials

The following supporting information can be downloaded at: https://www.mdpi.com/article/10.3390/polym18202459/s1, Table S1: Composition and pH of the film-forming solutions used for the preparation of chitosan–NaDES films; Table S2: Semi-quantitative FTIR peak-fitting analysis of the 1500–1650 cm^−1^ FTIR region of CS–DES_GA2 films containing different DES_GA2 contents; Figure S1: Appearance of the extract-containing film-forming solutions prior to vacuum filtration: 50DES_GA2_EN and 50DES_LA1_EN containing neem extract, and 50DES_GA2_EH containing hawthorn extract; Figure S2: Vacuum filtration of the DES_GA2 extract-containing film-forming solutions and appearance of the insoluble material retained on the filter; Figure S3: Peak-fitting analysis of the 1500–1650 cm^−1^ FTIR region for (a) 44DES_GA2, (b) 50DES_GA2, (c) 60DES_GA2, and (d) 70DES_GA2 films. Experimental spectra were baseline-corrected and fitted using three Gaussian components. The cumulative fitted curve represents the sum of the individual components; Figure S4: Visual appearance of CS – NaDES films with different DES_GA2 contents and plant extract incorporation; Figure S5: Representative stress – strain curves of CS – NaDES films containing 44–70 wt% DES_GA2 and of the neem-containing 50DES_GA2_EN film; Figure S6: Preliminary screening of the effect of aqueous DES_LA1 concentration on the total phenolic content (TPC) of hawthorn extracts. DES_LA1 corresponds to ChCl:LA at a 1:1 molar ratio. Water and 50% aqueous ethanol were used as reference extraction media. Different letters indicate statistically significant differences among extracts (*p* < 0.05). Figure S7: Representative images of the antibacterial activity of neem extract (Ex_EN_GA2), the corresponding extract-containing film (50DES_GA2_EN), and the extract-free film (50DES_GA2) against *E. coli* (left column) and *B. subtilis* (right column); Table S3: Principal values of thermal analysis for the studied NaDES; Figure S8: TG (solid) and DSC (dashed) curves for pure NaDES; Table S4: Experimentally obtained density and viscosity for the obtained NaDESs at different temperatures; Figure S9: Density and viscosity dependences with temperature for pure NaDES; Figure S10: FTIR spectra of neat DES_GA2. References [65,66,67] are cited in the Supplementary Materials.
