# Supplementary material for: Effect of ChCl:Glycolic Acid Molar Ratio and Plant Extract Incorporation on the Structure–Property Relationships of Chitosan Active Packaging Films
Source: Polymers (Basel). 2026 Oct 9;18(20):2459. doi: 10.3390/polym18202459 (PMC13708844; doi:10.3390/polym18202459)
Supplement: Supplementary file 1 [file polymers-18-02459-s001.zip › polymers-4598572-supplementary.pdf]

Effect of ChCl:Glycolic Acid Molar Ratio and Plant Extract Incorporation on the Structure – Property Relationships of Chitosan Active Packaging Films

Paul Stanescu[1], Adina Gavrila[1], Gabriela Isopencu[1], Ludmila Motelica[2,3], Adrian Ionut Nicoara[1,3], Sergiu Sima[1], Mihaela Bumbaru Dandes[1], Oana Ciocirlan[1]*, Ovidiu-Cristian Oprea[1,3,4]

[1]Faculty of Chemical Engineering and Biotechnologies, National University of Science and Technology Politehnica Bucharest, 132 Calea Grivitei, 010737, Bucharest, Romania
[2]Research Center for Advanced Materials, Products and Processes, National University of Science and Technology POLITEHNICA Bucharest, 313 Splaiul Independentei, 060042, Bucharest, Romania
[3]National Research Center for Micro and Nanomaterials, National University of Science and Technology POLITEHNICA Bucharest, 313 Spl. Independenței, 060042 Bucharest, Romania
[4]Academy of Romanian Scientists, 3 Ilfov Street, 050044 Bucharest, Romania

**Correspondence: oana.ciocirlan@upb.ro*

Table S1. Composition and pH of the film-forming solutions used for the preparation of chitosan–NaDES films.

Film acronym	Directly added NaDES (g)	mCS (g)	mNaDES/mCS	m water (g)	Total solids (% w/v)	CS (% w/v)	30% NaDES extract solution added (g)*	NaDES in film solids (wt%)	pH
44DES_GA2	0.42	0.53	0.79	49	1.9	1.0	0	44	5.7
50DES_GA2	0.5	0.5	1.00	49	2.0	1.0	0	50	5.1
60DES_GA2	0.6	0.4	1.50	49	2.0	0.8	0	60	3.9
70DES_GA2	0.7	0.3	2.33	49	2.0	0.6	0	70	3.4
50DES_GA2_ EN	0	0.5	1.00	49	2.0	1.0	1.67	50	4.3
50DES_GA2_ EH	0	0.5	1.00	49	2.0	1.0	1.67	50	4.2
50DES_LA1_ EN	0	0.5	1.00	49	2.0	1.0	1.67	50	4.2

*For extract-containing formulations, NaDES was supplied through the 30% (w/w) aqueous NaDES extract solution.

Table S2. Semi-quantitative peak-fitting analysis of the 1500–1650 cm^{-1} FTIR region of CS–DES_GA2 films containing different DES_GA2 contents.

Film	Component 1 position (cm^{-1})	Relative area (%)	Component 2 position (cm^{-1})	Relative area (%)	Component 3 position (cm^{-1})	Relative area (%)	R^2
44DES_GA2	1527.9	20.1	1573.7	70.9	1621.4	9.0	0.997
50DES_GA2	1528.0	20.6	1574.7	70.9	1623.6	8.5	0.996
60DES_GA2	1529.0	19.1	1577.1	72.7	1625.2	8.2	0.997
70DES_GA2	1529.2	16.1	1579.3	72.9	1625.6	11.1	0.993

The 1500–1650 cm^{-1} region was baseline-corrected and fitted using three Gaussian components under identical fitting conditions for all samples. Relative peak areas were calculated as the area of each fitted component divided by the sum of the areas of the three components. Because this spectral region contains overlapping contributions from amino, amide, and carboxylate groups, the fitted components were not assigned to individual vibrations. The analysis was therefore used for semi-quantitative comparison of spectral changes rather than for direct determination of the degree of amino group protonation.

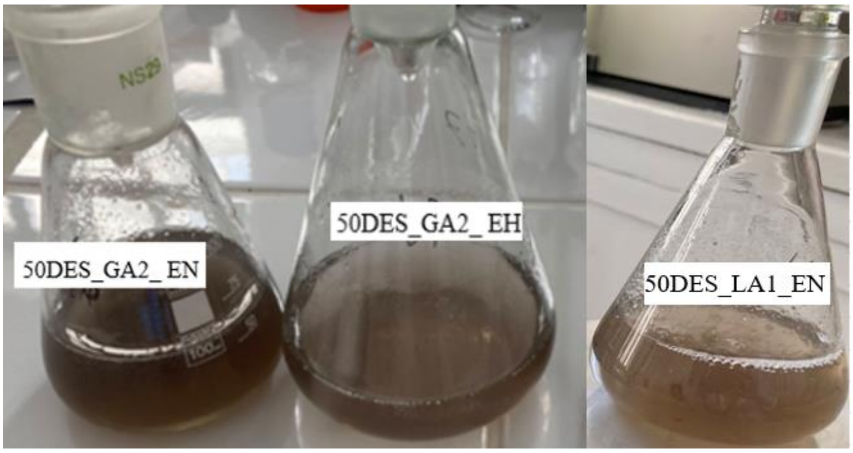

Figure S1. Appearance of the extract-containing film-forming solutions prior to vacuum filtration: 50DES_GA2_EN and 50DES_LA1_EN containing neem extract, and 50DES_GA2_EH containing hawthorn extract.

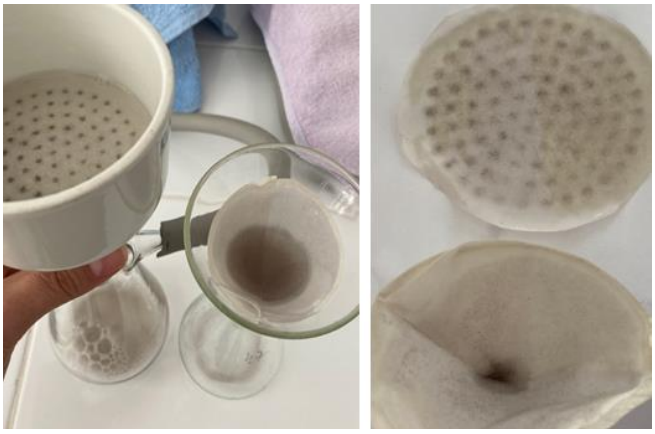

Figure S2. Vacuum filtration of the DES_GA2 extract-containing film-forming solutions and appearance of the insoluble material retained on the filter.

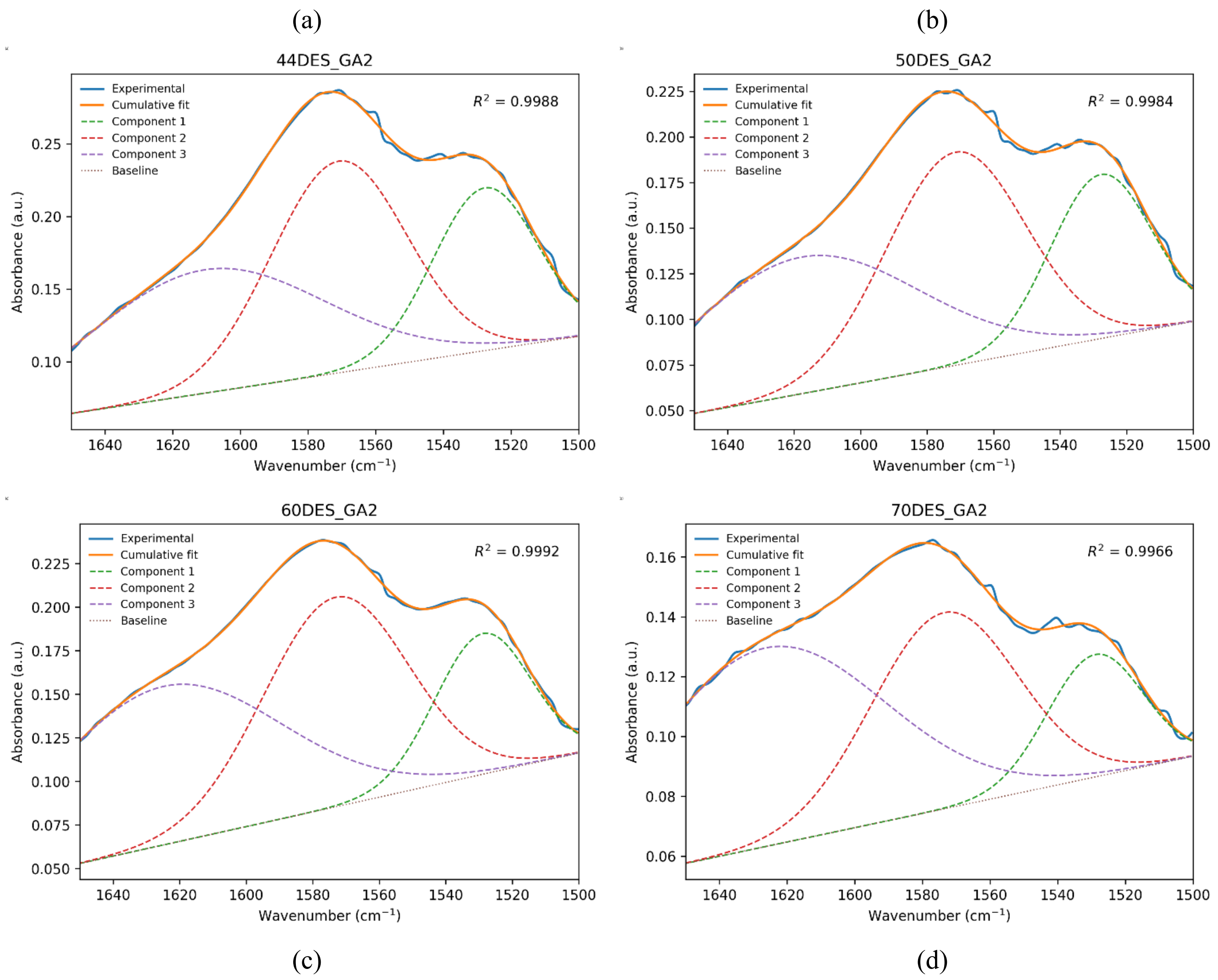

Figure S3. Peak-fitting analysis of the 1500–1650 cm^{-1} FTIR region for (a) 44DES_GA2, (b) 50DES_GA2, (c) 60DES_GA2, and (d) 70DES_GA2 films. Experimental spectra were baseline-corrected and fitted using three Gaussian components. The cumulative fitted curve represents the sum of the individual components.

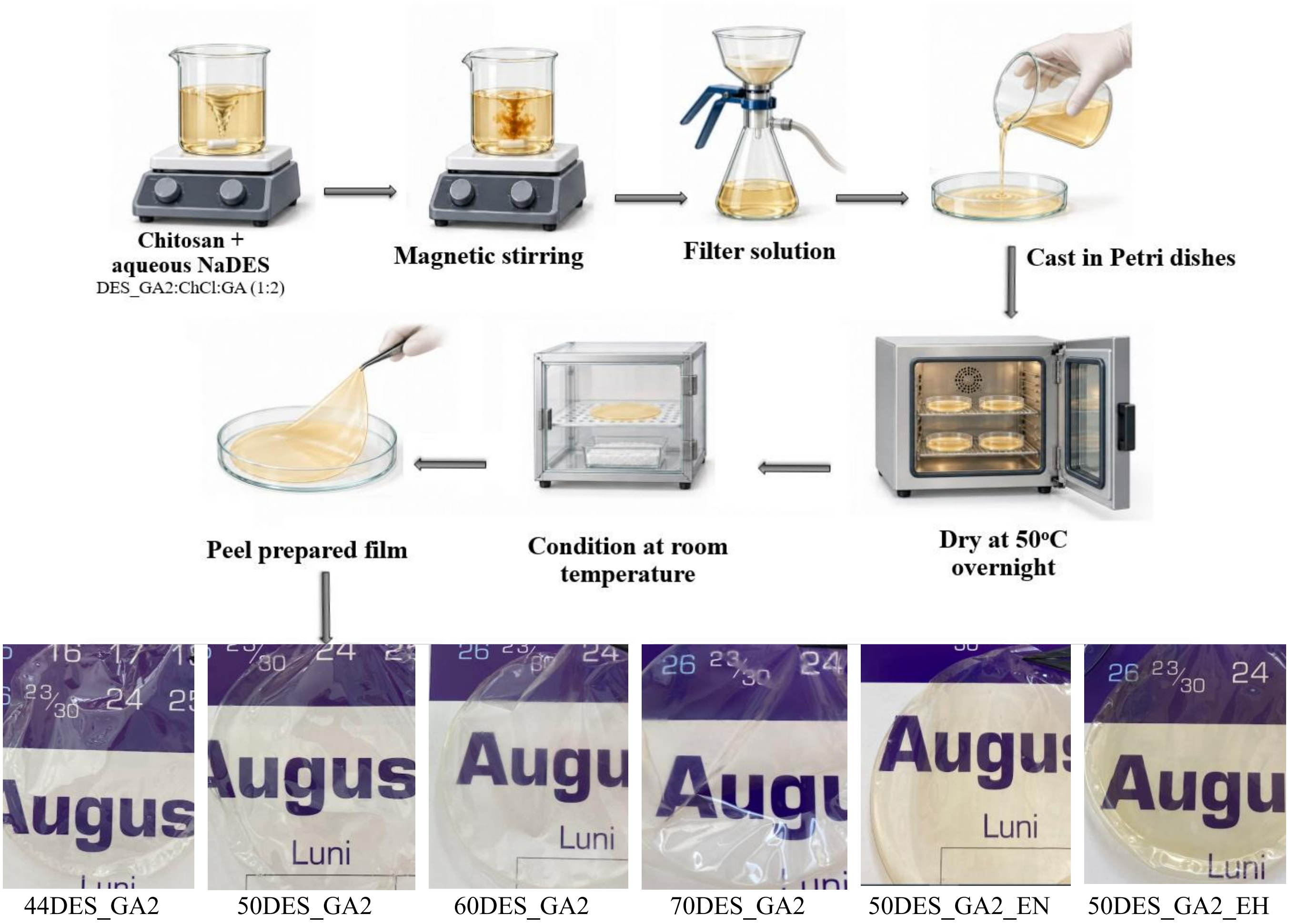

Figure S4. Visual appearance of CS–NaDES films with different DES_GA2 contents and plant extract incorporation.

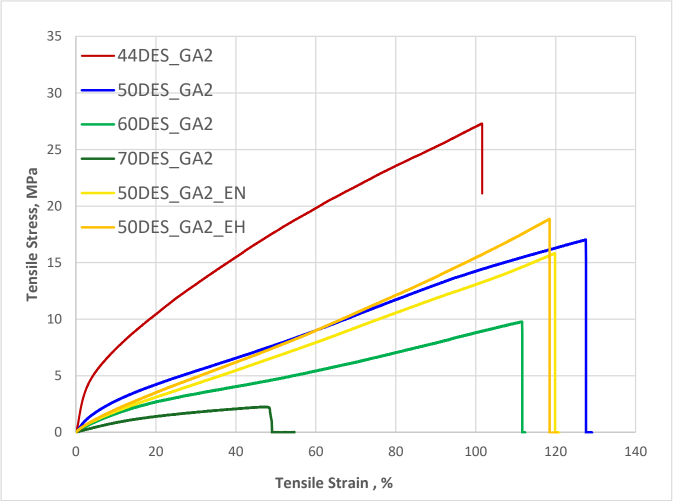

Figure S5. Representative stress–strain curves of CS–NaDES films containing 44-70 wt% DES_GA2 and of the neem-containing 50DES_GA2_EN film.

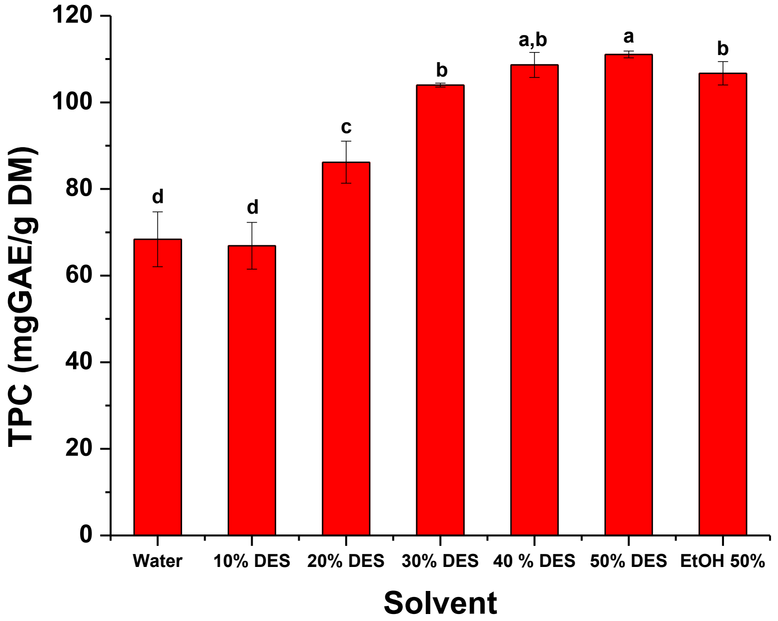

Figure S6. Preliminary screening of the effect of aqueous DES_LA1 concentration on the total phenolic content (TPC) of hawthorn extracts. DES_LA1 corresponds to ChCl:LA at a 1:1 molar ratio.
Water and 50% aqueous ethanol were used as reference extraction media.
Different letters indicate statistically significant differences among extracts ($p < 0.05$).

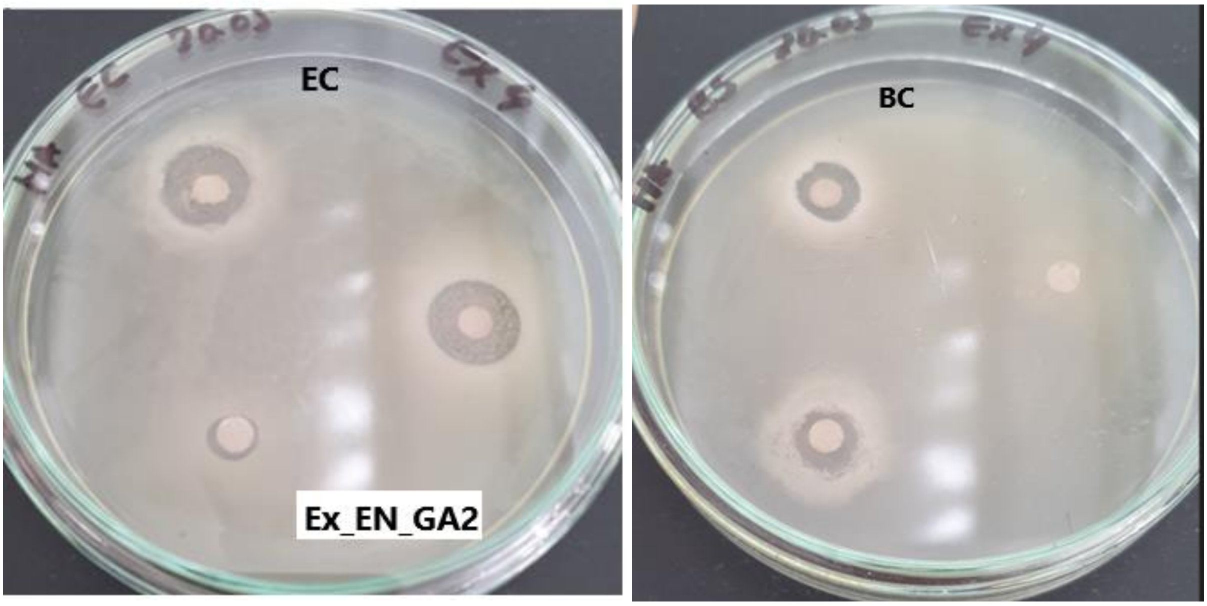

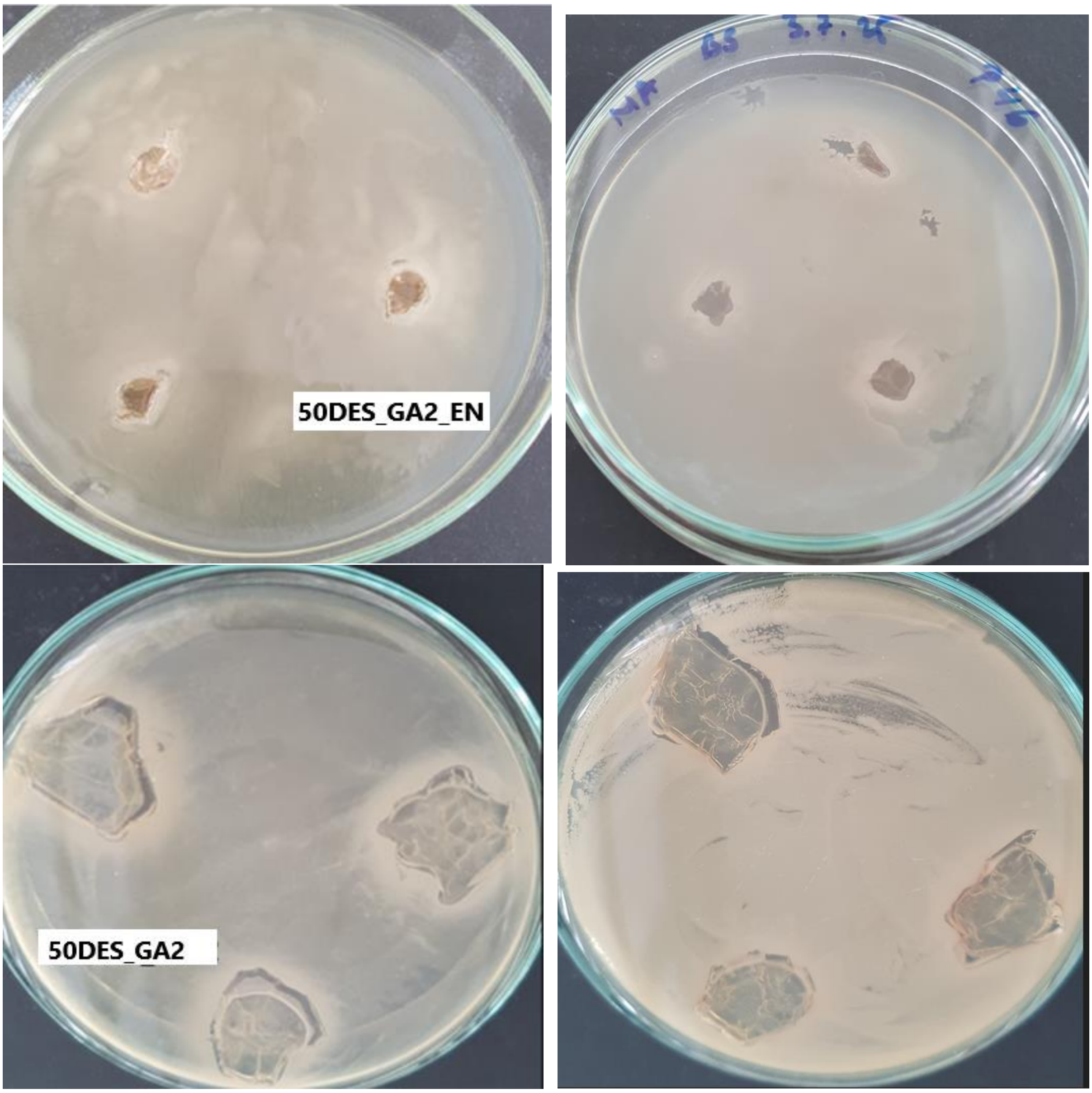

Figure S7. Representative images of the antibacterial activity of neem extract (Ex_EN_GA2), the corresponding extract-containing film (50DES_GA2_EN), and the extract-free film (50DES_GA2) against *E. coli* (left column) and *B. subtilis* (right column).

Characterization of pure NaDES

The thermal behaviour of DES_GA2 is presented in Table S3 and Figure S8. A small initial mass loss was observed at low temperature, attributable mainly to residual water and other volatile species. The first major thermal event occurred around 222°C, followed by the principal mass loss step associated with an endothermic event at approximately 272°C. A further high-temperature exothermic event was observed at approximately 467°C under the oxidative atmosphere employed. The onset of the main thermal degradation process is consistent with values previously reported for ChCl–GA systems prepared under similar conditions (Rodriguez Rodriguez et al., 2019). Overall, DES_GA2 showed adequate thermal stability over the temperature range relevant to the preparation and processing conditions employed in the present study.

Long-term stability of ChCl–GA deep eutectic solvents has also been reported by Jablonský et al. (Jablonský et al., 2018), supporting their potential for practical applications.

Table S3. Principal values of thermal analysis for the studied NaDES.

Sample	Endo I (°C)	Mass loss (%) RT-220°C	Exo I (°C)	Endo II (°C)	Mass loss (%) 220-280°C	Mass loss (%) 280-600°C	Exo II (°C)
DES_GA2	70.4	10.8	222.2	271.9	84.5	4.7	466.7

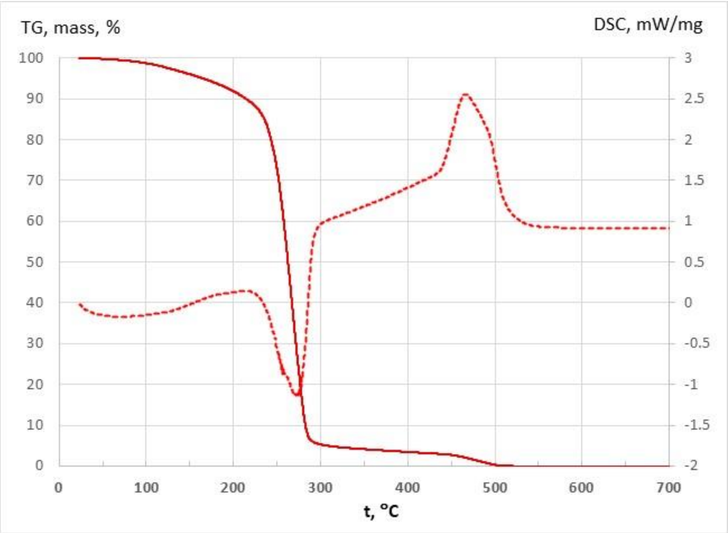

Figure S8. TG (solid) and DSC (dashed) curves for pure NaDES

The experimental density and viscosity data for DES_GA2 over the investigated temperature range are reported in Table S4 and Figure S9. The density decreased approximately linearly with increasing temperature, whereas viscosity showed a pronounced nonlinear decrease. At 298.15 K, DES_GA2 exhibited a density of 1.2400 g cm^{-3} and a viscosity of 280.0 mPa·s. The relatively high viscosity is consistent with the extensive intermolecular interactions characteristic of ChCl-based eutectic systems, particularly hydrogen-bonding interactions between the components (Ciocirlan et al., 2010).

Table S4. Experimentally obtained density and viscosity for the obtained NaDESs at different temperatures.

T/ K							
293.15	298.15	303.15	308.15	313.15	318.15	323.15	333.15
$\rho \cdot 10^{-3}$ / $kg \cdot m^{-3}$							
1.24322	1.24000	1.23678	1.23356	1.23046	1.22739	1.22429	1.21809
η / mPa·s							
		392.8	280.0	201.7	149.9	114.8	

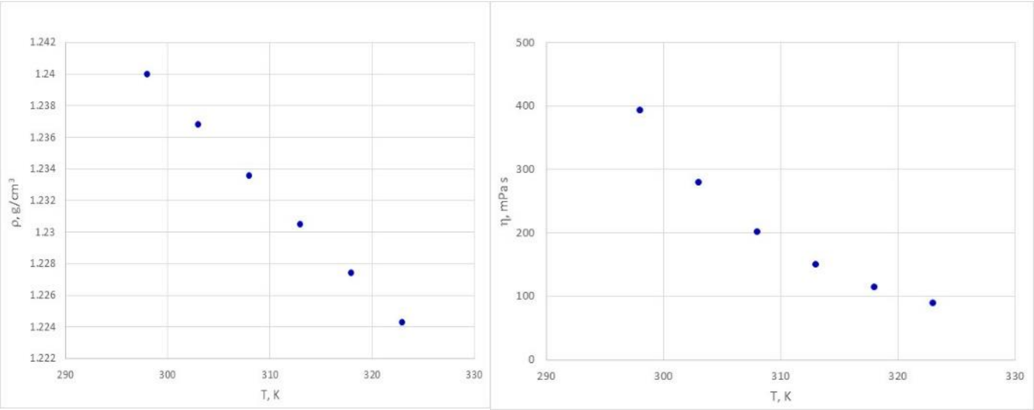

Figure S9. Density and viscosity dependence on temperature for pure NaDES.

The FTIR spectrum of DES_GA2 is shown in Figure S10. Comparison with the spectra of the individual components, ChCl and GA, previously reported by Ciocirlan et al., 2025, reveals changes consistent with the establishment of intermolecular interactions upon DES formation. In particular, changes in the carbonyl region and in characteristic ChCl bands indicate modification of the hydrogen-bonding environment in DES_GA2. Similar spectral changes have been reported for related ChCl–organic acid deep eutectic systems (Nazlı et al., 2025).

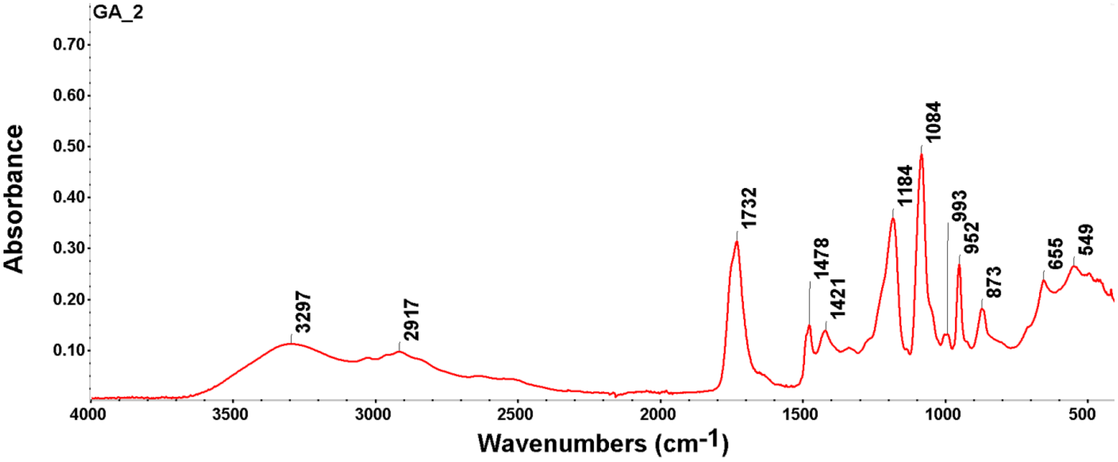

Figure S10. FTIR spectra of neat DES_GA2.

References

Ciocirlan, O., Gavrila, A., Isopencu, G., Motelica, L., Oprea, O.-C., Nicoara, A. I., Sima, S., & Stanescu, P. (2025). Formulation and Characterization of Chitosan Films Incorporating Hawthorn Polyphenolic Extracts via Natural Deep Eutectic Solvents. *Polymers*. https://doi.org/10.3390/polym17243250

Ciocirlan, O., Iulian, O., & Croitoru, O. (2010). Effect of temperature on the physico-chemical properties of three ionic liquids containing choline chloride. *Revista de Chimie*, *61*(8), 721–723.

Jablonský, M., Škulcová, A., Ház, A., Šima, J., & Majová, V. (2018). Long-term Isothermal Stability of Deep Eutectic Solvents. *BioResources*, *13*(4), 7545–7559.

Nazlı, F., Hasdemir, I., Uysal, E., Dursun, H. N., Gezici, U. O., Özçelik, D. Y., Burat, F., & Gürmen, S. (2025). Eco-Friendly Leaching of Spent Lithium-Ion Battery Black Mass Using a Ternary Deep

Eutectic Solvent System Based on Choline Chloride, Glycolic Acid, and Ascorbic Acid. *Minerals*, *15*(8), 782. https://doi.org/10.3390/min15080782

Rodriguez Rodriguez, N., van den Bruinhorst, A., B M Kollau, L. J., Kroon, M. C., Binnemans, K., & Vzw, S. (2019). Degradation of Deep-Eutectic Solvents Based on Choline Chloride and Carboxylic Acids. *ACS Sustainable Chem. Eng.*, *8*, 2025. https://doi.org/10.1021/acssuschemeng.9b01378
